# Training and education provided to local change champions within implementation trials: a rapid systematic review

**DOI:** 10.1186/s13012-025-01416-9

**Published:** 2025-02-05

**Authors:** Laura Jolliffe, Natasha A. Lannin, Stacy Larcombe, Brendan Major, Tammy Hoffmann, Elizabeth Lynch

**Affiliations:** 1 Department of Occupational Therapy, Peninsula Health, 2 Hastings Road, Frankston, VIC 3199 Australia; 2 Department of Occupational Therapy, School of Primary and Allied Health Care, Monash University, 47-49 Moorooduc Highway, Frankston, VIC 3199 Australia; 3 National Centre for Healthy Ageing, 2 Hastings Road, Frankston, VIC 3199 Australia; 4 Department of Neuroscience, Central Clinical School, Monash University, 99 Commercial Road, Prahran, Melbourne, VIC 3004 Australia; 5 Alfred Health, 55 Commercial Road, Prahran, Melbourne, VIC 3004 Australia; 6 Caring Futures Institute, College of Nursing and Health Sciences, Flinders University, Sturt North Sturt Rd, Bedford Park, Adelaide, SA 5042 Australia; 7 College of Medicine and Public Health, Flinders University, Adelaide, Australia; 8 Institute for Evidence-Based Healthcare, Faculty of Health Sciences and Medicine, Bond University, 14 University Drive, Robina, QLD 4226 Australia

**Keywords:** Implementation Science, Knowledge Translation, Rehabilitation, Evidence-Based Practice, Occupational Therapy

## Abstract

**Background:**

Translating research into clinical practice is challenging. One implementation intervention that supports translation is employment of a change champion. It is important to understand how individuals are prepared for the change champion role. This rapid systematic review aimed to identify the education, training, and support provided to individuals in change champion roles within implementation trials.

**Method:**

Rapid review approach. We searched the Scopus database to identify systematic reviews on champions, knowledge brokers, facilitators, and implementation support practitioners. The most recent reviews on each topic were screened to find eligible studies. To identify studies published after these reviews, we searched Medline, PsycINFO, OVID, CINAHL, ProQuest, SCOPUS, and EBSCO. We included randomised and cluster randomised controlled trials that reported on implementation interventions in healthcare settings involving a local change champion.

**Results:**

Fifteen cluster randomised controlled trials were included. Specific champion training was provided in 12 studies (80%), but none reported incorporating adult learning principles into their education program. Some form of post-training support was reported in 11 studies (73%). Only two studies included content on behaviour or organizational change in the champion preparation program. Most programs were not individualized, and details of training and support were poorly reported.

**Conclusions:**

Training needs and educational outcomes of change champions are poorly reported in implementation trials. Training tends not to align with adult learning. More rigorous development and reporting of programs to prepare change champions to support implementation of evidence in healthcare is recommended.

**Registration:**

PROSPERO registration number CRD42022368276.

**Supplementary Information:**

The online version contains supplementary material available at 10.1186/s13012-025-01416-9.

Contributions to the literature

- Our review highlights that the specific educational content provided to local change champions in implementation trials is often poorly detailed, which limits the ability to replicate successful interventions and understand their impact.
- Suggests incorporating established adult learning principles to enhance the effectiveness of training programs for local change champions, aiming to improve overall implementation outcomes.
- Emphasises the need for thorough reporting on the type and frequency of support provided to change champions, which is crucial for comprehending their role and enhancing the success of implementation efforts.

## Background

It is well established that implementing evidence-based practices in the clinical healthcare environment can be challenging [1–3]. One intervention commonly used to build implementation capacity is to employ or nominate individuals to support implementation within the clinical environment [4, 5]. Common across many implementation and scale-up frameworks [6, 7], these individuals often act in intermediary roles between researchers and front-line clinicians to facilitate implementation of evidence-based practices in healthcare [8, 9]. With many labels applied to these intermediary roles (at times used interchangeably), they include Knowledge Broker, Facilitator, Champion, Opinion Leader, Boundary Spanner, Coach, Consultant, Embedded Researcher, Technical Assistance Provider, and Implementation Support Practitioner [10, 11]. Hereafter, we will use the term *champion* to describe the people and their actions in this intermediary role. Consistently, these champions play an implementation role, and they ideally are: 1) internal to the recipient organisation; 2) have an intrinsic interest and commitment to implementing a change; 3) work diligently and relentlessly to drive implementation forward, even if those efforts receive no formal recognition or compensation; 4) are enthusiastic, dynamic, energetic, personable, and persistent; and 5) have the strength of conviction [12].

There is uncertainty about the effectiveness of the use of champions as a mechanism of behaviour change in healthcare. For example, the use of *facilitators* in a large cluster randomised controlled trial (cRCT) was not more effective than dissemination of guidelines for improving documentation of continence recommendations in residential aged care facilities [13], whereas in a systematic review, practice facilitation by a champion was associated with improved processes of care for people with chronic disease in North American primary care settings [14]. A comprehensive systematic review about the effectiveness of the subset champion group of *knowledge brokers* also reported conflicting findings from studies for changing knowledge, skills or policies and practice [15]. Further, a systematic review regarding the use of *champions* indicated that this strategy did not consistently improve individual healthcare professionals’ use of best practice, yet it did improve the use of best practice by systems and facilities [16]. Unfortunately, across these reviews the definition of the champion role differed, and there remains a need for a comprehensive review across the breadth of literature.

Previous reviews have focused on unpacking the core roles of champions to understand requirements regarding key knowledge, attributes [11], skills [17], and competencies [18]. Other research has focused on leadership and interpersonal skills required by change champions [19, 20]. There is a small but growing body of evidence about how to develop implementation capacity in champions, including the strategies and approaches used in implementation capacity building [5, 21] and the subsequent design of implementation training [22–25] being applied to the champion role. However, details about the type of training provided to champions to prepare and upskill them for their role in implementation trials are unclear. Therefore, the aims of this rapid review were to identify the education, training, and support provided to individuals in change champion roles within implementation trials. To achieve this, the following research questions were posed:

1. How were champions engaged at each involved trial site (including process of champion identification and numbers)?
2. What proportion of included trials provided formal training to site champions?
3. What type of training was provided, including content, method of delivery, training frequency, learner goals, and knowledge outcomes?
4. What maintenance strategies were employed following initial education, including training and support during the trial, as well as once the trial was complete?

## Method

A rapid systematic review of implementation trials was conducted. Rapid reviews are literature reviews in which components of the systematic review process are simplified, while maintaining transparent and reproducible methods [26, 27]. Applied in various ways to reduce the time taken to synthesize findings, rapid reviews frequently limit the literature search by date, language and to published literature only, and limit personnel requirements during screening and data extraction [26]. This review was conducted to inform the design of our own champion training for a national implementation trial (currently underway, ANZCTR trial registration: ACTRN12623000608662), with the rapid review approach most appropriate for timely completion. Informed by the Cochrane Rapid Reviews Method [28], our team selected the most recently published systematic and scoping reviews as primary data sources to identify potential trials for inclusion in this review. This was considered most appropriate approach to abbreviate the search process, given that these existing systematic reviews yield the same trials of interest required to answer our research questions [27]. To identify RCTs published in the years after the systematic reviews, we replicated an included systematic review search. Our rapid review was pre-registered with the International Prospective Register of Systematic reviews (PROSPERO: CRD42022368276).

### Searches

#### Part A: Search for systematic review and/or scoping reviews

As a strategy for identifying eligible RCTs, we initially searched for systematic reviews or scoping reviews that addressed the effectiveness of, or important attributes for: *champions, knowledge brokers*, *facilitators*, and *implementation support practitioners.* The search was conducted in Scopus up to November 2023 (search strategy in supplementary file 1). Additionally, authors contacted known experts in the field of implementation science to identify systematic reviews and trials which could potentially be eligible (names provided in supplementary file 2). Search results were independently reviewed by EL & LJ for inclusion. The most recently published systematic and/or scoping review related to each of the four terms were selected as the primary data source. Whilst recency of publications does not equate to quality, this was deemed an acceptable approach to reduce the time it takes to identify RCTs of interest [29]. In an attempt to mitigate the risk of review quality, we included multiple systematic reviews / scoping reviews. Two reviewers (LJ and SL or BM) screened the reference lists of included systematic review sources to identify all potentially eligible RCTs and cRCTs.

#### Part B: Search to identify recently published RCTs

To identify eligible RCTs published after date of the latest included systematic review (2022) [16], we replicated the search strategy published by Santos and colleagues [16] in Medline, PsycINFO, OVID, CINAHL, ProQuest, SCOPUS, EBSCO. We consulted with a research librarian to confirm search terms (nothing was altered). We date limited our search from 2020- search date, assuming that relevant RCTs before this time were identified from the systematic reviews. Only RCTs published in English were included in our review, as our team did not have access to translation services, and this approach aligns with the accepted methodology for rapid reviews to ensure timely completion [28] (see supplementary file 3 for search strategy).

### Study selection

No publication date limits were set for implementation trials. Trial inclusion criteria were:

- RCT or cRCT design
- conducted in healthcare setting:inpatient, outpatient, or community;
- study aims included implementation of evidence by health professionals;
- included a change champion,local to the study site, as a standalone or part of an implementation strategy
- English language

Studies identified through the systematic search were uploaded to Covidence systematic review software for screening and management (Veritas Health Innovation, Melbourne, Australia. Available at www.covidence.org). Titles and abstracts were screened by one reviewer (SL), all excluded studies were reviewed by a second reviewer (LJ or EL). Two reviewers (LJ or EL and SL) independently screened full texts to determine inclusion. All disagreements were resolved through discussion and consensus from the full research team.

### Study quality assessment

The inclusion of a risk of bias rating is recommended by the Cochrane handbook for rapid reviews [29]. The PEDro scale is widely used in healthcare systematic reviews and has recognized validity and reliability [30–32]. The PEDro scale scores 10 items: random allocation; concealed allocation; similarity at baseline; subject blinding; therapist blinding; assessor blinding; > 85% follow up for at least one key outcome; intention-to-treat analysis; between-group statistical comparison for at least one key outcome; and point and variability measures for at least one key outcome. Items are scored as either present (1) or absent (0) and a score out of 10 is obtained by summation. PEDro scores of 0–3 are considered ‘poor’, 4–5 ‘fair’, 6–8 ‘good’, and 9–10 ‘excellent’ [31, 33]. One reviewer (LJ) completed the PEDro scale for each included study.

### Data extraction and outcomes of interest

A data extraction template was created and piloted. Approximately 20% of data extraction was independently extracted by two reviewers (LJ and SL) and discussed to ensure a consistent data extraction process. A single reviewer (SL or LJ) then extracted the remaining data, which was checked by a second reviewer (LJ or EL) for accuracy. The decision to have a single reviewer extract data was a pragmatic one within our rapid review to accelerate the review process; acceptable within a rapid review design [29]. Data extracted included study details such as trial design and intervention components based on the Template for Intervention Description and Replication (TIDieR) framework [34]. This included specific details of the active intervention components such as dose, strategies, training, and support. Data related to trial effectiveness, including policy outcomes, implementation outcomes, service outcomes, and patient-level outcomes were also extracted. In addition, all implementation strategies were mapped to the nine categories described by Powell et al. [6] which are: Use evaluative and iterative strategies; provide interactive assistance; adapt and tailor to context; develop stakeholder interrelationships; train and educate stakeholders; support clinicians; engage consumers; utilize financial strategies; and, change infrastructure [6]. The data extraction form is provided in supplementary file 4.

Where there were missing data or a limited description of training, the corresponding authors of the study were contacted by reviewers (LJ, EL) for this information, and/or separately published process evaluations were reviewed for further information where available.

### Data analysis

Review findings were synthesised narratively [35, 36]. The results were compared and consolidated through consensus between two reviewers (LJ, EL) and then discussed with all authors.

## Results

**Fig. 1:**
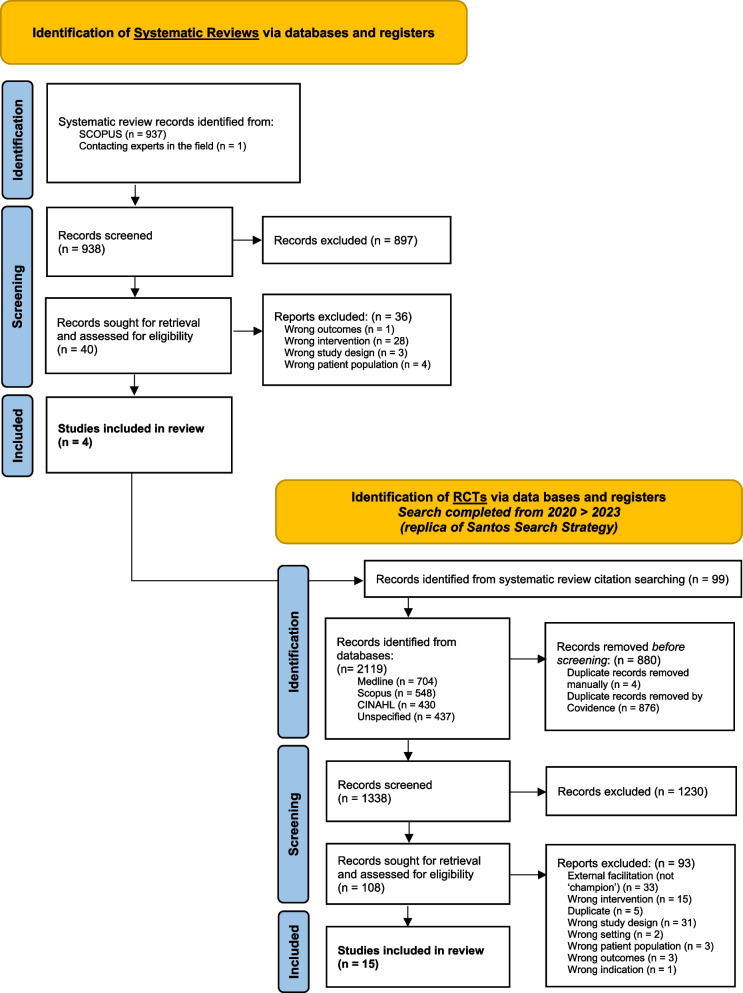
PRISMA flow diagram [38] outlining the review's selection process

The *Part A* search (Supplementary file 1) identified four systematic and/or scoping reviews: Santos et al. [16] for *champions;* Bornbaum et al. [15] for *knowledge brokers,* Cranley et al. [8] and Moussa [37] for *effectiveness of facilitation* and *facilitation strategies*. An additional systematic review, Albers [17] for *implementation support practitioners,* was also included based on expert recommendation. As per the registered search method, trials extracted from these systematic reviews were added to trials identified from the *Part B* search (Supplementary file 3). After duplicate removal and title/abstract screening, 108 full-text studies were assessed for eligibility, of which 15 studies met our inclusion criteria and were included in our review. The primary reason for exclusion being either wrong study design i.e., not using RCT or cRCT design or using an external implementation support person and not a local site champion as defined for our review. Refer to Fig. 1 PRISMA flowchart [38], and supplementary file 5 for excluded papers and exclusion reasons.

Two corresponding authors, Bunce [39] and Mudge [40] were contacted for further information about their respective trials. Bunce and Gold met with two reviewers (LJ and EL) via videocall to provide information about training content and champion selection. Mudge and colleagues were unable to share information at the time, given the pending publication of their study-related process evaluation. In the time since, this process evaluation has been published and incorporated into our review [41].

### Study characteristics

Of the 15 included studies, all were cRCTs with randomisation at the site or ward level. Studies were conducted in Australia (*n* = 3 studies), the United Kingdom (*n* = 3), the USA (*n* = 5), Kenya (*n* = 1), Malawi (*n* = 1), Canada (*n* = 1) and Europe (*n* = 1). Seven studies measured implementation outcomes only [13, 39, 42–46], two studies measured only patient outcomes [40, 47] and six studies measured both implementation and patient outcomes [48–53]. Seven measured implementation outcomes and reported improvements in one or more measures [42, 43, 46, 48, 51–53]. Of the eight studies that measured patient outcomes, only three studies reported an improvement [48, 51, 53]. See Tables 1 and 2 for full detail of included studies. Most studies (12, 80%) were of ‘good’ quality and 3 studies (20%) were excellent [54]. Most studies lost scores for not clearly describing randomization allocation concealment or blinding processes. Full details of quality assessments are included in Supplementary File 6.

**Table 1:** Study characteristics of included trials (*n* = 15)

**Study ID**	**Title**	**Country of study**	**Study design**	**Study setting**	**Aim of study**	**Study start date**	**Study end date**	**Total number of health professionals (discipline, number)**	**Patient group involved (clinical area, number)**	**PEDro Scale Score (/10)**
Acolet 2011 [48]	Improvement in neonatal intensive care unit care: a cluster randomised controlled trial of active dissemination of information	England (United Kingdom)	Cluster RCT	Inpatient hospital	To assess whether an evidence-based innovative active knowledge transfer strategy was more likely to lead to changes in policy and practice than the passive dissemination of the report, the slide package and the neonatal position statement on a website	2006	2007	87 clinicians (profession not specified)	94 units for 355 babies born between 22 and 26 weeks (169 from 45 units in the active arm, and 186 from 49 units in the control arm)	9
Ayieko 2011 [42]	A multifaceted intervention to implement guidelines and improve admission paediatric care in Kenyan district hospitals: a cluster randomised trial	Kenya	Cluster RCT	Inpatient hospital	Study hypothesis: That the quality of paediatric inpatient care at district hospitals in Kenya can be improved by an intervention over 18 months comprising evidence-based guidelines, didactic training, job aides, supervision, feedback and facilitation of local problem solving	July 2006	May 2009	72 health workers (profession not specified)	Paediatric inpatients *N* = 2135 at baseline, *N* = 2315 at 18 months	7
Bailey 2021 [45]	Comparison of Two Methods for Implementing Comfort Care Sets in the Inpatient Setting: a Cluster Randomised Trial	United States	Cluster RCT	Veteran Affairs Medical Centres (VAMCs)	To evaluate and compare two methods of delivering the BEACON intervention utilizing the established infrastructure of VA Palliative Care Consult Teams (PCCT): a traditional implementation approach using a teleconference and an enhanced implementation approach utilizing in-person, train-the-champion workshops, both to review educational materials and prepare PCCT members to be onsite clinical champions	March 2015	April 2019	132 providers from PCCTs	Medical record audit deceased veterans *N* = 6,491 *N* = 46 VAMCs randomised, *N* = 88 providers from 23 VAMCs received teleconference *N* = 44 providers from 23 VAMCs received in-person workshop training	6
Bunce 2020 [39]	Process Evaluation: Lessons learned about the effective operationalization of champions as an implementation strategy: results from a qualitative process evaluation of a pragmatic trial Main paper: Does increased implementation support improve community clinics’ guideline-concordant care? Results of a mixed methods, pragmatic comparative effectiveness trial	United States	Cluster RCT	Community clinics	To compare the effectiveness of 3 implementation strategies (low, medium, high intensity) to support community health centres implement guideline-concordant cardioprotective prescribing for patients with diabetes	Aug 2015	July 2018	Not reported. Minimum of 18 (main paper), but unsure total number of staff involved in each arm of the study	29 health clinics: Arm 1-low intensity; 9 clinics (*n* = 3849 patients) Arm 2- medium intensity support; 11 clinics (*n* = 5098) Arm 3- high intensity support; 9 clinics (3370) Natural comparison, 33,639 patients)	7
Cadilhac 2022 [43]	A Stepped-Wedge Cluster-Randomized Trial to Improve Adherence to Evidence-Based Practices for Acute Stroke Management	Australia	Cluster RCT	Inpatient hospital	To test the effectiveness of a co-designed multifaceted intervention (STELAR: Shared Team Efforts Leading to Adherence Results) directed at hospital clinicians for improving acute stroke care tailored to the local context using feedback of national registry indicator data	May 2017	Sept 2018	144 clinicians attended 18 intervention workshops (nurses, allied health, medical staff, managers and students) in nine hospitals (clusters) participated	Acute stroke patients: *n* = 3147	8
Duclos 2022 [51]	Effect of monitoring surgical outcomes using control charts to reduce major adverse events in patients: cluster randomised trial	France	Cluster RCT	Inpatient hospital	To determine the effect of introducing prospective monitoring of outcomes using control charts and regular feedback on indicators to surgical teams on major adverse events in patients	Jan 2017	Dec 2018	Not reported	*N* = 159,688 patients underwent digestive tract surgery *N* = 75,047 intervention *N* = 80,315 control	8
Johnston 2007 [46]	One-on-One Coaching to Improve Pain Assessment and Management Practices of Pediatric Nurses	Canada	Cluster RCT	Paediatric hospital	To determine if one-on- one coaching based on audit with feedback and “think-aloud” interactions with an opinion leader could change attitudes and knowledge about pain in children and improve pain practices, specifically assessment and management by pediatric nurses with pediatric inpatients	2001	2003	*N* = 141 nurses who worked at least three full shifts per week on inpatient units	Paediatric patients in the care of a study nurse *N* = 1,602 chart audits (985 chart audits on 306 children in the preintervention phase and 617 on 158 children in the postintervention phase)	6
Llewelyn 2023 [52]	Antibiotic review kit for hospitals (ARK-Hospital): a stepped-wedge cluster-randomised controlled trial	United States	Stepped-wedge cluster RCT	Inpatient hospital	The antibiotic review kit (ARK) program for hospitals aimed to develop and evaluate a multifaceted behaviour change intervention to safely reduce antibiotic use in acute general medical inpatients	Sept 2017	Nov 2017	Prescribers (i.e. Doctors and Nursing Practitioners)	Pseudonymised electronic health records of adult (age ≥ 16yrs) non-elective general or medical inpatients Acute general medical admissions: *n* = 7,160,421	7
Middleton 2019 [50]	Nurse-Initiated Acute Stroke Care in Emergency Departments	Australia	Cluster RCT	Inpatient hospital	We aimed to evaluate the effectiveness of an intervention to improve triage, treatment, and transfer for patients with acute stroke admitted to the emergency department	July 2013	Sept 2016	Not reported	Patients admitted to the stroke unit via ED. *N* = 1597	9
Mudge 2022 [40, 41]	Main paper: Effect of a Ward-Based Program on Hospital-Associated Complications and Length of Stay for Older Inpatients: The Cluster Randomized CHERISH Trial Process evaluation: Implementing a ward-based program to improve care for older inpatients: process evaluation of the cluster randomised CHERISH trial	Australia	Cluster RCT	Inpatient hospital	To implement and evaluate a ward-based improvement program ("Eat Walk Engage") to more consistently deliver age-friendly principles of care to older individuals in acute inpatient wards	Oct 2016	Oct 2017	Not reported	Inpatients aged 65 years. *N* = 539	6
Puchalski Ritchie 2021 [47]	Process evaluation of an implementation strategy to support uptake of a tuberculosis treatment adherence intervention to improve TB care and outcomes in Malawi	Malawi	Cluster RCT	Community clinic	Our objectives were to refine a previously piloted TB treatment adherence intervention, designed to give LHWs the knowledge and skills needed to address common causes of TB treatment non-adherence [15, 16], and to evaluate its effectiveness in improving TB treatment outcomes	May 2016	Feb 2018	169 lay health workers	74 HCs and 798 tuberculosis patients	7
Resnick 2021 [53]	Testing the Implementation of Function-focused Care in Assisted Living Settings	United States	Cluster RCT	Assisted Living Facilities	The purpose of this study was to evaluate the Function-Focused Care for Assisted Living Using the Evidence Integration Triangle (FFC-AL-EIT) intervention	Not reported	Not reported (+ 18-months)	Not reported	85 sites and 794 residents Participants: average age of 89 years, 71% female and 97% white	7
Resnick 2021 [49]	Implementation of the Evidence Integration Triangle for behavioral and psychological symptoms of dementia (EIT-4-BPSD) in care communities	United States	Cluster RCT	Inpatient care community / RACF	To test an implementation strategy, the Evidence Integration Triangle for behavioural and psychological symptoms of distress in dementia (BPSD) (EIT-4-BPSD), for assisting staff in the use of evidence-based behavioural approaches for BPSD	Apr 2016	June 2021	193: nurses and other staff working at the care communities	Not stated	8
Rycroft-Malone 2012 [44]	A pragmatic cluster randomised trial evaluating three implementation interventions	United Kingdom	Cluster RCT	Inpatient hospital	What is the most effective implementation strategy for the uptake of evidence-based recommendations about peri-operative fasting?	Jan 2006	June 2009	19 acute hospitals	*N* = 3,505 duration of fasting observations were recorded	8
Seers 2018 [13]	Facilitating Implementation of Research Evidence (FIRE): an international cluster randomised controlled trial to evaluate two models of facilitation informed by the Promoting Action on Research Implementation in Health Services (PARIHS) framework	United Kingdom	Cluster RCT	Nursing homes	Extend knowledge of facilitation as a process for getting research evidence into practice by testing the effectiveness of and evaluating the contribution two different models of facilitation can make to implementing evidence-based urinary continence recommendations into practice	2010	2013	Not reported	Long term care. *N* = 2313 patient records (residents)	9

*RACF * Residential Aged Care Facility, *RCT * Randomised control trial

**Table 2:** Details about implementation strategy of each included study

Study ID	Overall effectiveness of the trial	Length of implementation period:	Implementation target (what recommendation/practice/behaviour)	Implementation strategies used:	Implementation strategy category/ categories (refer to Powell 2015; Waltz 2015)
Acolet 2011 [48]	Policy outcomes: no change Implementation outcomes: Improvement in delivery of 1 of 3 recommended practices (delivery of trunk in plastic bag) (RR = 1.27; 95% CI 1.01 to 1.60; *p* = 0.04) Patient outcomes: improvements in 1 of 1 outcome (core temperature on admission) (mean difference = 0.29 °C; 95% CI 0.22 to 0.55; *p* = 0.03)	3 months: 2 doses, ad hoc support Change measured over 12-months	To improve policy and practice in preterm baby care Recommended practice: 1. All intubated babies (< 27 weeks) should receive surfactant within an hour of birth (as early as compatible with safety) 2. At all births < 27 weeks gestational age, the following should be called and be present before the baby is delivered: a consultant paediatrician or a middle grade practitioner and a Senior House Officer or an Advanced Neonatal Nurse Practitioner 3. Core temperature on admission to NICU should be at 36 °C (taken electronically) 4. Trunk delivered in a plastic bag to avoid hypothermia	Control arm: Dissemination of research report; slides; information about newborn care position statement Active arm: Dissemination of research report; slides; information about newborn care position statement, offer to become ‘regional ‘champion’ (attend two workshops, support clinicians to implement research evidence regionally), or attend one workshop, promote implementation of research evidence locally The intervention components included: Dissemination of materials: research report; slides; information about newborn care position statement Site champion: offer to become ‘regional ‘champion’ (attend two workshops, support clinicians to implement research evidence regionally), or attend one workshop, promote implementation of research evidence locally Follow up support: contact and support via email and telephone over the following 2–3 months	• Train and educate stakeholders (distribute educational materials) • Develop stakeholder interrelationships (identify and prepare champions) • Support clinicians (remind clinicians)
Ayieko 2011 [42]	Policy outcomes: N/A Implementation outcomes: improvements in delivery of10/18 indicators •Average assessment score (0.05 to 0.54 95% CI; *p* = 0.03) •Severe malaria episodes with twice daily quinine maintenance dose, (25.1 to 60.2 95% CI; *p* = 0.001) •Severe malaria episodes with quinine daily dose 40 mg/kg (-12.9 to -0.2 95% CI; *p* = 0.045) •Gentamicin prescriptions with once daily dose (8.04 to 26.1 95% CI; *p* = 0.004) •Gentamicin prescriptions with daily dose, 4 mg/kg (-11.9 to -1.59 95% CI; *p* = 0.019) •Correct intravenous fluid prescription (10.9 to 48.9 95% CI; *p* = 0.008) •Adequate oxygen prescriptions (7.32 to 62.8 95% CI; *p* = 0.021) •Pneumonia episodes with a severity classification (9.87 to 67.3 95% CI; *p* = 0.017) •Malaria episodes with a severity classification, (26.2 to 78.0 95% CI; *p* = 0.003) •Dehydration episodes with a severity classification (4.27 to 24.6 95% CI; *p* = 0.013 Patient outcomes: N/A	18 months: Multiple doses, ongoing support Change measured at 18 months	Primary effectiveness measures were 14 process indicators measured on paediatric admissions aged 2–59 months Process indicators: 1. Child's weight documented, 2. Child temp documented 3. Average assessment score 4. Severe malaria episodes with twice daily quinine maintenance dose, 5. Severe malaria episodes with quinine loading, 6. Severe malaria episodes with quinine daily dose 40 mg/kg 7. Gentamicin prescriptions with once daily dose, 8. Gentamicin prescriptions with daily dose,4 mg/kg, 9. Gentamicin prescriptions with daily dose 10 mg/kg, 10. Correct intravenous fluid prescription, 11. Adequate oxygen prescriptions, 12. Pneumonia episodes with a severity classification, 13. Malaria episodes with a severity classification, 14. Dehydration episodes with a severity classification Outcome indicators: 1. Vitamin A administered on admission, 2. Provider initiated HIV testing among unknown HIV, 3. Age appropriate documentation of immunisation status 4. Mean proportion discharge counselling tasks performed	Control group: 6-monthly assessment survey and written feedback, 1.5 days of didactic education, dissemination of evidence-based clinical guidelines and job aides (drug doses charts, fluid and feed prescription charts, structured admission records) Intervention group: 6-monthly assessment survey and written and face-to-face feedback, 5.5 day training program, dissemination of evidence-based clinical guidelines and job aides (drug doses charts, fluid and feed prescription charts, structured admission records); external supervisory process; local facilitator to drive basic quality improvement; support via telephone every 1–2 weeks The intervention components included: Survey and feedback: 6-monthly survey to assess hospital followed by written feedback to both groups, face-to-face feedback at intervention sites only Training: training aimed at 32 health workers of all cadres approximately 6–10 weeks after baseline surveys in intervention hospitals, Dissemination of materials: provision of clinical practice guidelines and job aides, External support: an external supervisory process Site champion: full-time local facilitator (a nurse or diploma-level clinician) responsible for promoting guideline use and on-site problem solving Follow up support: Supervision visits (by research team) were approximately two to three monthly, telephone support 1–2 weekly	• Use evaluative and iterative strategies (audit and feedback) • Provide interactive assistance (facilitation) • Develop stakeholder interrelationships (identify and prepare champions) • Train and educate stakeholders (train the trainer, distribute educational materials) • Change infrastructure (change record systems)
Bailey 2021 [45]	Policy outcomes: N/A Implementation outcomes: no difference between two groups adjusted odds ratio = 1.18 (95% CI 0.74 to 1.89) Patient outcomes: NA	4-months	To assess improvement of process-of-care endpoints between in-person train the champion workshop and traditional teleconference methods Primary recommendation: improvement of process-of-care endpoints (active opioid order at time of death, DNR order in place, advance directive, family present) in VAMCs	Arm 1: Teleconference • Aligned with standard VA training methods • No limit for number of PCCT members attending • Delivered 80 min teleconference guided by PowerPoint presentation providing didactic instruction and facilitated group discussion Arm 2: In-Person Train-the-Trainer Workshop • Two or three PCCT members attended a 2-day in-person workshop, where the training team provided a standardized curriculum, including review of the education package; case-based coaching to build care plans using the CCOS; hands-on computer lab experiences using the CCOS; and coaching to become champions (trainers and change agents) • Small group discussions occurred to review experience with CCOS and compare care plans. PCCT members also reviewed teaching concepts, techniques and materials for training other providers	• Provide interactive assistance (facilitation, technical assistance) • Adapt and tailor to the context (tailor strategies) • Develop stakeholder interrelationships (identify and prepare champions) • Train and educate stakeholders (train the trainer, distribute educational materials) • Change infrastructure (change record systems)
Bunce 2020 [39]	Policy outcomes: N/A Implementation outcomes: no significant differences between study arms In the adjusted difference-in-difference model, arms 1 and 2 experienced significantly greater increases in statin prescribing (compared to other indicators) over the study period than the comparison CHCs did: • 5% greater in arm 1 (RR 1.05; 95% CI 1.01–1.08) • 6% greater in arm 2 (RR 1.06; 95% CI 1.03–1.09) Prescribing gains in arm 3 were no different from those in the comparison CHCs (RR 1.02; 95% CI 0.99–1.06) Patient outcomes: N/A	4 years: multiple doses, ongoing support Change measured over 5 years	Aim: The study (SPREAD-NET: Study of Practices Enabling Implementation and Adaptation in the Safety Net) hypothesized that more intensive implementation support would lead to greater improvements in the targeted outcomes (support the adoption of guideline-concordant cardioprotective prescribing in community health centres, for patients with diabetes) Primary recommendation: patients with diabetes who were indicated for cardioprotective medications to be prescribed statins and ACE/ARBs	Natural comparison: a set of similar clinics (*n* = 137) as a natural comparison group for the use in quantitative analyses Arm 1: Implementation toolkit Arm 2: Implementation toolkit, in-person training, and training webinars Arm 3: Implementation toolkit, in-person training, training webinars and practice facilitation Arm 1 1) Stakeholder relationships: Identify and prepare champions (one or more champion to lead implementation activities and liaise with study team) 2) Distribute educational materials: Develop and distribute educational materials (implementation toolkit covering how to use CVD bundle components and tips on practice change) 3) Train and educate: Conduct ongoing trainings (annual webinars on the CVD bundle) [electronic health record-based tools; basic webinar; discussion with clinicians and using monthly feedback report] Arm 2: Above interventions [1-3] plus: 4) Train and educate: conduct educational meetings/use train the trainer strategies (2 days in person training focused on the use of the CVD bundle and implementation toolkit) 5) Train and educate: Conduct ongoing trainings (quartile webinars with content based on training needs) Arm 3: Above interventions [1–5] plus: 5) Facilitation: Practice facilitation from an implementation specialist. Offered practice facilitation > = 2 onsite visits in the first year; > = 3 visits over the 2nd and 3rd years. Provided coaching on tools, tailored problem-solving support for barriers, clinical questions fielded by RN practice facilitator and site clinical champion	• Provide interactive assistance (facilitation, clinical supervision) • Develop stakeholder interrelationships (identify and prepare champions) • Train and educate stakeholders (train the trainer, ongoing training, distribute educational materials)
Cadilhac 2022 [43]	Policy outcomes: N/A Implementation outcomes: effective for selected clinical indicators (primary outcome; 6% improvement 95% CI 3–30) Improvements in 4 of 10 clinical indicators, reduction in 1 clinical indicator: •Treated in a stroke unit (OR 1.43 (1.13–1.80 95%CI) •Received thrombolysis within 60 min of hospital arrival (OR 0.37 (0.15–0.89 95% CI) •Discharged on antithrombotic medications (OR 1.90 (1.45–2.49 95% CI) •Discharged on lipid lowering medication (OR 1.42 (1.14–1.77 95% CI) •Discharged to the community with a care plan (OR 1.46 (1.11–1.92 95% CI) Patient outcome: N/A	2 months: multiple doses, ongoing support Change measured over 12-months	To improve adherence to clinical indicators for acute stroke care The clinical indicators applicable to all patients were: 1. treated in a stroke unit 2. having a swallow screen/assessment prior to oral intake 3. mobilized on the same or next day following admission 4. being prescribed antihypertensive medication 5. being prescribed antithrombotic medication 6. receiving a care plan when discharged The clinical indicators for patients with ischemic stroke were 1. treated with intravenous thrombolysis 2. receiving this drug within 60 min of hospital arrival 3. receiving antiplatelet medication within 48 h of arrival 4. being discharged on lipid lowering medication Each site selected 2–3 priority indicators for action plan Primary outcome: composite outcome of (2 or 3) indicators selected for action plans Secondary outcomes: adherence to each indicator above	Stepped-wedged design: The STELAR implementation intervention had 2 main stages comprising 4 components Audit and feedback: using data from the AuSCR for 10 clinical indicators aligned to the national stroke care standards. This was provided as an educational outreach event involving direct contact between implementation facilitators and clinicians. This was set up as a workshop conducted as a data review meeting via 1-h videoconference Training: The second workshop was conducted for 2 h within 1 month of the first workshop. This comprised of an in-person educational component focused on the evidence supporting the indicators prioritized in the first workshop, followed by a discussion among the multidisciplinary team members regarding those modifiable barriers within their hospitals that could be addressed for the prioritized indicators Second workshop: Education provided by study facilitator and developed a site-specific action plan. Site nominated local change champion Remote ongoing support via telephone or email for 2 months	• Use evaluative and iterative strategies (audit/feedback, assess for readiness and identify barriers and enablers) • Provide interactive assistance (facilitation) • Develop stakeholder interrelationships (e.g. identify and prepare champions) • Train and educate stakeholders (distribute educational materials)
Duclos 2022 [51]	Policy outcomes: NA Implementation outcomes: Significant reduction in the frequency of deterioration signals (0.60, 95% confidence interval 0.37 to 0.96; *P* = 0.03) and a significant increase in the frequency of improvement signals (3.89, 1.40 to 10.83; *P* = 0.009) in intervention versus control hospitals Patient outcomes: Composite primary outcome of major adverse events within 30-days after surgery absolute risk reduction of 0.9% (95% CI 0.4% to 1.4%) between groups (in favour of intervention group).: Significant reduction in major adverse events (-0.84, 95%CI 0.77 to 0.92), patient death (-0.84, 0.71 to 0.99), intensive care stay (0.85, 0.76 to 0.94)	2-year implementation period	To facilitate implementation of control charts to monitor surgical outcomes and reduce major adverse events Primary outcome: composite of major adverse events including inpatient death, intensive care stay (at least two nights in intensive care or five nights in critical care), reoperation (open or laparoscopic), and/or severe complications (cardiac arrest, pulmonary embolism, sepsis or surgical site infection) within 30 days after surgery Secondary outcomes: 1. Frequency of signal detection related to deterioration or improvement in surgical outcomes on control charts 2. Compliance with control chart implementation programme on outcomes	Intervention Group: Champion duo was established at each site, responsible for conducting meetings to review control chart and maintaining a logbook to record changes in care processes. Champion duos from each hospital met during three one day training sessions held at intervals of eight months. Simulated roleplay and feedback from participants at these sessions were aimed at providing the skills needed to use the control charts appropriately, leading review meetings for effective cooperation and decision making, identifying variations in special causes, and devising plans for improvement Control Group: Usual care, that is, no specific intervention	• Use evaluative and iterative strategies (feedback) • Train and educate stakeholders (ongoing training) • Provide interactive assistance (role-play and facilitation) • Develop stakeholder interrelationships (organise clinician implementation team meetings) • Adapt and tailor to the context (tailor strategies) • Change infrastructure (change record systems and mandate change)
Johnston 2007 [46]	Policy outcomes: N/A Implementation outcomes: the nurses' knowledge and attitudes about pain in children [F(1,791) = 106.3, *p* < .0001] as well as the assessment and management of children's pain increased in the coaching group (no between group statistics reported) Patient outcomes: NA	18 months	To improve paediatric nurses' (1) knowledge and attitudes of pain in children, (2) rate of pain assessment, (3) rate of administering ordered analgesics, and (4) rate of using nonpharmacological interventions during painful procedures	Control • Audits (four audits per nurse) was conducted monthly Intervention • Coaching session (delivered by the coach-champion) every 2 weeks for 6 months • Audit at experimental sites and continued until each nurse received at least 10 coaching sessions • Resource kit- including ‘sourcebook’ *Coaching Sessions:* • Before each meeting, the coach received audits of the study nurses’ charts for review • Coach recognised aspects of pain assessment and management conducted and what facilitated those actions; as well as barriers that may have contributed to inadequate or lack of action • Solutions were sought from study nurses via a think-aloud process, to encourage clinical reasoning. The steps identified in this method were cue acquisition, hypothesis generation, cue interpretation, intervention, rationale and hypothesis evaluation	• Use evaluative and iterative strategies (audit/feedback. Identify barriers) • Adapt and tailor to the context (tailor strategies, promote adaptability) • Develop stakeholder interrelationships (identify and prepare champions) • Train and educate stakeholders
Llewelyn 2023 [52]	Policy outcomes: NA Implementation outcomes: Reduction in monthly antibiotic defined-daily-doses per acute general medical admission following intervention (-4.8%, 95%CI -9.1 to -0.2, no p value reported) Patient outcomes: Non-significant reduction in all-cause mortality within 30-days of admission (-2.7%, 95%CI -5.7 to 0.3, no p value reported)	24-months	To reduce unnecessary antibiotic administration and overuse in adult acute general medical inpatients Two co-primary outcomes: • defined-daily-doses (DDD) of antibiotics per acute/general medical admission (superiority) • 30-day mortality post-admission (non-inferiority) Both outcomes were assessed by estimating the immediate effect and the sustained year-on-year effect. Intervention effects were assessed by interrupted time series analyses within each site	Intervention: • Local site champion • Intervention was a decision aid embedded in hospital prescription process, prompting prescribers to clarify the level of diagnostic uncertainty at antibiotic initiation by classifying infection risk as possible or probable; then ceasing prescription if a clear indication for ongoing antibiotic treatment could not be established at 48-72 h • Online training to motivate and support use of the decision aid • Provision of implementation guidance (including audit and feedback tools) • Patient leaflet (i.e. handout) Control: standard care	• Use evaluative and iterative strategies (audit/feedback) • Develop stakeholder interrelationships (identification and prepare champions) • Train and educate stakeholders (ongoing training, distribute educational materials)
Middleton 2019 [50]	Policy outcomes: N/A Implementation outcomes: no change Patient outcomes: no change	5–7 months: (workshops + bedding down with reminders) Multiple doses, ongoing/adhoc support Change measured after 3-month bedding down period	To improve triage, treatment, and transfer for patients with acute stroke admitted to the emergency department Primary outcome: death or dependency at 90 days Secondary outcomes: 90-day functional dependency, health status, 11 ED quality of care measures as below 1. Triage to Australasian Triage Scale category 1 or 2 2. Assessment for tPA eligibility 3. Temperature readings on admission and 4-hrly in ED 4. Formal blood glucose level sent to laboratory on admission to ED 5. Finger prick glucose readings on admission and 6hrly whilst in ED 6. Maintenance of nil by mouth or receipt of a swallow screen or assessment within 24 h of ED admission 7. Maintenance of nil by mouth until receipt of a swallow screen or assessment 8. Receipt of a swallow screen or assessment within 24 h of ED admission 9. Given oral fluid or food prior to the first swallow screen or swallow assessment 10. Given oral medical prior to the first swallow screen or swallow assessment 11. Prompt (< 4 h) stroke unit transfer	Control group: did not receive clinical protocols or additional support Intervention group: Nurse-initiated evidence-based clinical protocols developed by stroke and ED clinical experts for triage, treatment (assessment for eligibility for tPA; administration of tPA to eligible patients; fever, sugar, swallowing management), and prompt transfer to a stroke unit Multi-disciplinary workshops: 2 × 1-h face-to-face workshops with ED and stroke clinicians delivered 6–18 weeks apart to identify local barriers to implementation and identify site champions (Workshop 1) and develop site-specific action plan (Workshop 2) Interactive and didactic education: 20 min PowerPoint presentation and 10 min discussion; 8 min video developed by an academic ED nurse clinician/opinion leader National opinion leader in stroke care present at workshop Site clinical champions from ED and Stroke Unit Reminders: ED posters; lanyard cards; site visits (every 3 months), teleconference (every 3-months with clinical champions and site coordinator), email (monthly + reactive), telephone support (reactive)	• Use evaluative and iterative strategies (identify barriers and enablers) • Develop stakeholder interrelationships (identify and prepare champions, organise clinician implementation team meetings) • Train and educate stakeholders (distribute education materials) • Support clinicians (remind clinicians)
Mudge 2022 [40, 41]	Policy outcomes: N/A Implementation outcomes: N/A Process outcomes: no significant difference in length of stay or facility discharge Patient outcomes: no change	6 months: multiple doses, ongoing follow-up Change measured over 3 years	To consistently deliver age-friendly principles of care (nutrition and hydration, mobility, and meaningful cognitive and social engagement), to older individuals in acute inpatient wards Primary outcome: incidence of any hospital-associated complications including delirium, hospital-associated disability, incontinence, pressure injuries and falls; length of stay Secondary outcomes: incidence of individual hospital-associated complications, facility discharge, 6-month readmission, all-cause readmission	Local facilitator: employed 2 days/week. Coordinated monthly 1-h group meetings with the ward multidisciplinary team; identified local barriers and enablers; facilitated the planning and implementation of small-scale improvement cycles as agreed by the team to support the program aims; identified delegation tasks suitable for the additional part time health care assistant; audit and feedback of structured process measures related to the improvement aims; liaising with higher level decision makers when needed Formation of workgroup: The multidisciplinary working group included key stakeholders and champions (eg, nurse unit manager, nurse educator, physiotherapist, dietitian, and occupational therapist) who met for 1 h per month and worked with the facilitator and other staff as required toward iterative improvements Additional resources: a half-time multi-professional assistant employed and trained on each ward who could have tasks delegated from any team member	• Use evaluative and iterative strategies (audit/feedback, assess for readiness and identify barriers and enablers) • Provide interactive assistance (internal and external facilitation) • Train and educate stakeholders (distribute education materials) • Develop stakeholder interrelationships (identify and prepare champions, organise clinician implementation team meetings) • Support clinicians (create new clinical teams)
Puchalski Ritchie 2021 [47]	Policy outcomes: N/A Implementation outcomes: N/A Patient outcomes: no change	4 months: multiple doses, ad hoc follow-up Change measured over 12 months	Improve adherence to TB treatment Outcome: TB treatment success	Educational outreach: Peer-led educational outreach by lay health workers trained as peer trainers Cascade training: 8 cascade training sessions each a minimum of 60 min over 4–5 months Educational resources: clinical support tool, laminated flip chart outlining treatment Peer support network: small telephone stipends provided quarterly to peer trainers to support development of a peer support network among peer trainers Support from research team: available via phone when initiated by peer trainers	• Develop stakeholder interrelationships (identify and prepare champions, peer network support) • Train and educate stakeholders (train the trainer, ongoing training, distribute educational materials)
Resnick 2021 [53]	Policy outcomes: policy assessment supporting function focused care (mean difference 1.13, *p* ≤ 0.003) between groups at 12-months Implementation outcomes: there was an increase in adoption and use of function-focused care interventions in the treatment sites from 63% at baseline to 68% at 4 months and 90% at 12 months Patient outcomes: Intervention group had less decline in function (mean difference 6.9; *p* ≤ .001), more function-focused care (mean difference 5%, *p* = .012)	18-month period Resident outcomes measured at baseline, 4 months and 12 months Setting outcomes obtained at baseline and 12 months	To deliver Function Focused Care to address persistent functional decline and increased sedentary time among assisted living residents intervention on facility (environment and policy assessments and service plans) and resident outcomes (function, physical activity and performance) Facility-level outcome: Environment and policies supporting function-focused care and service plans Resident-level outcome: Function and physical activity at 4 and 12 mo post implementation of the intervention	Intervention Group: • Identification of a site champion (any member of staff who expressed a desire to work with research team and make a practice change • Environment and Policy Assessments: Research Nurse Facilitator and Champion evaluated environment and policies within setting to consider how to support function-focused care and identify potential barriers. Sites also given activity resources ($50 value) to use with residents • Education of Direct Care Workers: Standardised PowerPoint presentation on function-focused care delivered to direct-care workers, families and residents. Multiple choice knowledge test to ensure receipt of information • Establishing Function-Focused Care Service Plans for Residents: Research Nurse Facilitator worked with the Champion to evaluate resident’s capability and establish service plans that incorporated functional and physical activities to the resident’s preference • Mentoring and Motivating: Research Nurse Facilitator worked with the Champion to implement techniques that motivate direct care workers, residents, and families to engage in function-focused care activities. Positive reinforcement, encouragement and observation completed by Champion to ensure function-focused care strategies were implemented Control Group: • Function-Focused Care Education only, which involved providing the same education as intervention groups	• Use evaluative and iterative strategies (identify barriers and enablers) • Provide interactive assistance (facilitation) • Adapt and tailor to the context (tailor strategies, promote adaptability) • Develop stakeholder interrelationships (identify and prepare champions) • Train and educate stakeholders (train the trainer, ongoing training, distribute educational materials) • Support clinicians (remind clinicians) • Engage consumers (involve patients/consumers and family members) • Utilise financial strategies (funding activity resources) • Change infrastructure (change physical structure and equipment)
Resnick 2021b [49]	Policy outcomes: no change Implementation outcomes: no change for all outcomes at 4 months or 12 months Patient outcomes: no change in resident outcomes at 4 months or 12 months except smaller proportion of residents in control group sites used anticonvulsant medication at 12 months (*p* = .01)	12 months: multiple doses, monthly follow-up for 12 months Change measured at 4 months, 12 months, 18 months	To improve adherence to evidence-based behavioural approaches for BPSD by staff working in residential aged care facilities (quality of care interactions, person-centred approaches, pain, function, use of psychotropic medications, quality of life) Organisational outcome measures: Environment Assessment, Policy Assessment for Person-Centered Management of BPSD; Evidence of Person-Centered Approaches for BPSD in Care Plans; Quality of Interaction Residential outcome measures: function, depression, agitation, resistiveness to care, use of psychotropic medications, pain, quality of life	Control group: Staff education about BPSD using PowerPoint presentation in 30-min sessions, provided in preferred format for residential aged care facility (e.g. face-to-face, webinar, conference call) Intervention group: • Identification of a site champion (any member of staff who expressed a desire to work with research team and make a practice change in the residential aged care facility. Site champion to encourage staff, provide feedback of performance • Initial staff education about BPSD using PowerPoint presentation in 30-min session, provided in preferred format for residential aged care facility (e.g. face-to-face, webinar, conference call) • Monthly meetings with stakeholders, site champion and research facilitator to identify site-specific goals and discuss progress towards site-specific goals and use of evidence-informed behavioral approaches for reducing BPSD • Supported to adjust and refine implementation strategies as needed • Education of staff by research team on use of person-centred approached for BPSD • Initial meeting 2–4 h, ongoing meetings 1–2 h. Weekly emails to Stakeholder team with BPSD titbits, motivational activities (contests with other sites)	• Provide interactive assistance (external facilitation) • Adapt and tailor to the context (tailor strategies, promote adaptability) • Develop stakeholder interrelationships (identify and prepare champions, organise clinician implementation team meetings) • Train and educate stakeholders (distribute education materials) • Support clinicians (remind clinicians)
Rycroft-Malone 2012 [44]	Policy outcomes: N/A Implementation outcomes: no change Patient outcomes: N/A	6 months: Multiple doses, no follow-up Change measured over 27 months	To increase adherence to peri-operative fasting times / increase duration of fluid fast prior to induction of anaesthesia Primary outcome: duration of fluid fast prior to induction of anaesthesia	Trusts were randomised to one of 3 interventions: Group one (*n* = 7): standard dissemination of a guideline package Group two (*n* = 6): standard dissemination of a guideline package + web based educational package encouraged by a local opinion leader Group three (*n* = 6): standard dissemination of a guideline package plus plan-do-study-act completed by a local champion Dissemination of materials: A copy of the RCN/RCA guideline (including overview of the guideline development process and those involved, recommendations, algorithm poster, and audit criteria); A patient version of the guideline; A PowerPoint presentation outlining some principles of guideline implementation Education: web-based educational package (The web-based resource was inter-active, incorporating educational tools such as self-check tests, working through clinical scenarios, and a patient digital story). championed by an opinion leader(s) Opinion leader: The web-based resource was championed by opinion leaders working in participating surgical areas Quality improvement approach: A dedicated champion with relevant clinical and/or managerial experience was identified by each site’s key contact. Champions had a one-day training session. All trusts received their individual baseline mean food and fluid fasting times at the beginning of the intervention phase	• Use evaluative and iterative strategies (audit and feedback) • Provide interactive assistance (e.g. facilitation) • Develop stakeholder interrelationships (e.g. identify and prepare champions) • Train and educate stakeholders (distribute education materials)
Seers 2018 [13]	Policy outcomes: N/A Implementation outcomes: no change Patient outcomes: N/A	24 months: multiple doses, ongoing support	The primary outcome was the documented percentage compliance with continence recommendations produced by the fourth International Consultation on Incontinence at 6, 12, 18, 24 months These recommendations included the resident should be actively screened for incontinence (5 components); a detailed assessment should be carried out (15 components); an individualised treatment plan should be in place (13 components); and a specialist referral should be made if needed (1 component)	Control group: Dissemination of educational materials: urinary continence recommendations; PowerPoint presentation on implementation sent to site head Intervention group 1: Dissemination of educational materials: urinary continence recommendations; PowerPoint presentation on implementation sent to site head Preparing internal facilitators: 3-day residential program – knowledge, skills, action planning Allocation of time: 10 days to work locally on the implementation and evaluation of recommendations Supporting facilitators to develop/embed skills: 12 half-days for monthly teleconferences and self-directed study Intervention group 2 Dissemination of educational materials: urinary continence recommendations; PowerPoint presentation on implementation sent to site head Preparing internal facilitators: 5-day residential program: knowledge, skills, action planning Allocation of time: 20 days to work on the local implementation and evaluation of the recommendations Supporting facilitators to develop/embed skills: 24 half-day learning groups via teleconferencing 12 half-days for self-directed study	• Provide interactive assistance (facilitation) Develop stakeholder interrelationships (identify and prepare champions) • Train and educate stakeholders (ongoing training, distribute education materials)

The duration of implementation interventions of the included studies varied greatly, ranging from two months up to four years. All studies used multiple implementation interventions within their overall strategy, with some interventions delivered on at least two occasions. All studies included interventions to develop stakeholder interrelationships by identifying and frequently preparing champions, and all studies provided educational materials. Other commonly used interventions were training and education which was provided in 14 studies (93%), with 7 of these (47%) using multiple education interventions. Interactive assistance was provided in 10 studies (67%), and evaluative and iterative interventions, such as audit and feedback or assessing barriers and enablers, were used in 9 studies (60%).

### Champion identification and engagement

All studies which provided details of the number of champions involved (12, 80%) engaged either one or two champions per site [13, 39, 40, 43–45, 47, 48, 50–53]. Champions were often site selected with desired attributes recommended by the research team [13, 39, 41, 43, 44, 46], however six studies did not report how the champion was selected. Champions were from mixed disciplines in five studies [39, 40, 43, 45, 51], from nursing backgrounds in four studies [13, 42, 46, 50], and from a non-clinical background in one study [52]. The years of clinical experience of champions was only reported in one study (≥ 4 years) [41], despite this, most studies referred to their champions as ‘clinical leads’ or ‘accepted opinion leaders’ implying a degree of experience or seniority. See Tables 3 and 4 for details.

**Table 3:** Details of site champion in included studies (*n* = 15)

Study ID	How was the champion identified?	Discipline of site champion	Years of clinical experience of champion	Champion training learner goals? and outcomes?	Details of training for implementation champion (e.g. how many champions/site, length of training, components of training package, who delivered training):	Details of support provided to implementation champion (ongoing and follow-up):	Enough details from paper to map above to Adult learning theory?
Acolet 2011 [48]	Not reported. "Lead clinician" was the site champion	Not reported- likely nurse	Not reported	Not reported	Name given to implementation champion: Regional champion Number of champions per site: 1 per site Length of training: Two whole-day workshops (one week apart) Components of training: 1. Educational material: A report which identified variations in standards of care that might have contributed to death in preterm babies born at 27- or 28-weeks’ gestation 2. Educational material: Informed about recommendations and the position statement on early care of the newborn available on the BAPM website, and sent an accompanying slide set specifically tailored for different audiences to aid discussions at local hospital clinical governance meetings 3. Workshop 1: provided regional champions with time to explore: (a) the theory and practice of NHS organisational change; (b) the role of champions as leaders; (c) behaviour change principles and the human dimension of changes and (d) how to develop practical skills to effect and sustain change in order to support health care staff in their workplace 4. At workshop 2, a week later, the regional champions and trainers, were joined by consultants and senior nurses from the active units to explore: (a) the research evidence in specified clinical areas summarised in lectures from national clinical leaders; (b) benchmarking of individual policies and practices and (c) introduction to tools and practice of change. The clinical leads were then asked to determine actions to: (a) develop responses to suggested areas of changes locally and (b) the support and processes needed to achieve these changes 5. After the workshops, the trial team distributed preliminary benchmarking information to allow participating units to see how practices in their own centres compared to practices in England overall Who delivered training: Workshops were supervised by trainers with expertise in organisational change	The research team also facilitated further contacts and support via email and telephone over the following 2–3 months	Not reported
Ayieko 2011 [42]	Not reported aside from “hospital selected”	Non-physician health worker. Most were nurses or "diploma-level clinician"	Not reported	Not reported	Name given to implementation champion: Local facilitator Number of champions per site: Not stated Length of training: 5.5-day training program extending WHOs emergency triage assessment and treatment approach to include management in line with clinical practice guidelines Components of training: modules or learning objectives were not reported on specifically Who delivered training: "External supervisory process" was provided by the research team. Limited details provided about trainer’s background	Supervisory activities: The implementation team conducted an additional 10–12-h training session in two intervention hospitals and two to three small group sessions of 2–4 h in all four intervention hospitals over the 18-month intervention period No other follow up was provided	No
Bailey 2021 [45]	Not reported	Most (60%) were prescribing providers (physicians, nurse practitioners) and 40% non-prescribing (RNs, pharmacists, mental health providers) and 8% from other services	Not reported	Not reported	Name given to implementation champion: Clinical champions Number of champions per site: 1–2 champions per site Length of training: 2-day in-person training Components of training: Arm 1: Teleconference group • BEACON Comfort Care Intervention: A multimodal approach which included didactic information on how to identify and care for patients who are dying in the hospital setting, the Comfort Care Order Set (CCOS), pocket cards, and other tools and training materials needed to train staff at their own hospital • Pre-implementation preparation: communication with administrative leaders, PCCT members and Information Resource Management representative; and provided technical assistance for CCOS in computerised patient record system Arm 2: In-person group • 2-day workshop: Interactive teaching on comfort care, case-based coaching, hands-on experience with CCOS, teaching to become trainers and change agents (with manual) • Biweekly conference calls with trainers to provide consultation for 3-months Who delivered training / disseminated material / provided support: Research Team	Arm 1: Availability of training team throughout 4-month implementation as required Arm 2: During 4-month implementation period, regularly scheduled bi-weekly conference calls were available for trained workshop participants to discuss local implementation	No
Bunce 2020 [39]	Site-identified champion Research team suggested (but require) selecting champions with enthusiasm about quality improvement, credibility, and influence at the clinic	Mix of: Physicians, nurses, admin staff, pharmacist, practice managers	Not reported	Not reported	Name given to implementation champion: Clinical champions Number of champions per site: 1–2 champions (each champion looked after between 1–3 sites) Length of training: 2 days in person training (arm 2 + 3) Components of training: not provided Who delivered training / disseminated material / provided support: Research Team (including practice facilitator) Arm 1 1) Identify and prepare champions (one or more champion to lead implementation activities and laisse with study team) and provided with description of their role Arm 2: 4) Train and educate: conduct educational meetings/use train the trainer strategies (2 days in person training focused on the use of the CVD bundle and implementation toolkit) 5) Train and educate: Conduct ongoing training (quartile webinars with content based on training needs) Arm 3: Above interventions plus: 5) Facilitation: Practice facilitation (PF) visits included meetings with point people, clinician champions, and clinic leadership, interviews with clinic employees to understand clinic functioning and capacity, and training on the guidelines underlying the CVD bundle. At initial visit, PF also spoke at staff/provider meetings	Arms 1–2 didn’t receive follow up support Arm 3: Practice facilitator provided virtual coaching tailored to each organisation’s needs, including monthly emails with study point people, webinars for clinic staff, and connecting staff to other resources (e.g., technical support). A second visit occurred at all arm 3 sites in March–May 2016; these visits content varied in response to clinic needs	No
Cadilhac 2022 [43]	Local clinical leaders elected by their site following X2 workshops Criteria: Anyone from the multidisciplinary stroke team who had responsibilities in delivering the selected clinical indicator to patients	The nominated change champions were predominantly nurses (71.4%) or allied health (6.5%; 9.5% being pharmacists)	Not reported	Not reported	Name given to implementation champion: change champions Number of champions per site: One champion per site Length of training: Champion did not receive any specific training Components of training: N/A Who delivered training / disseminated material / provided support: The research facilitator provided this training, who had a background in nursing and project management and had undertaken specific, formal training at a three-day course in knowledge translation methods by national leaders in this field	The research facilitator for the workshops provided ongoing remote support for a period of 2 months via telephone or e-mail to help facilitate implementation of the identified strategies as required	No
Duclos 2020 [51]	Not reported	Surgeon and another surgical team member (surgeon, anesthetist, or nurse)	Not reported	Not reported	Name given to implementation champion: Champion partnerships or champion duos Number of champions per site: 1 duo per site (2 people) Length of training: 3 × one-day training sessions held at intervals of 8 months Components of training: link to supplementary material although only available in French https://shewhart.univ-lyon1.fr/icap_website/view/2300 Who delivered training / disseminated material / provided support: Research team	Nil reported follow-up	No
Johnston 2007 [46]	Meetings held on the ward over a 4-week period to introduce the study, invite participation and obtain consent Nurse Education Department and Pain Services identified coaches. Individuals were eligible if they were (1) respected by peers, (2) viewed as a leader, (3) knowledgeable of pain in children, (4) interested in research, and (5) able to be released from their usual position to take a part-time role as a coach	Paediatric Nurse	Not reported	Not reported	Name given to champion: Coach Number of champions per site: Not reported Length of training: 2-day workshop, followed by 2-weekly follow-up coaching sessions Components of training: 2-day workshop focused on coaching session interactions • First day consisted on information about role, familiarising them with the sourcebook, and modifying the sourcebook's content or format based on feedback from the coaches • Second day was devoted to role playing of coach–nurse interactions. Process of interaction and information from nurse’ audit provided a think-aloud strategy to assist the nurses in explaining why a particular action or nonaction was taken. Based on what was expressed, the coach would provide evidence-based information • Resource kit developed at each site for coach to use as a reference for themselves and participating nurses • Resource material reviewed during training workshops with the coaches Who delivered training / disseminated material / provided support: Research Team	Investigators of the research team were available to the coach for consultation	No
Llewelyn 2023 [52]	Not specifically reported Described as “a local representative willing to lead intervention implementation and provide study data”	Microbiology/ Infectious Diseases, Acute Medicine or Pharmacist	Not reported	Not reported	Name given to champion: Champion Number of champions per site: 1 per site (implied from text) Length of training: Champion specific training was not specified Components of training: Not reported Who delivered training / disseminated material / provided support: Not reported	Not reported	No
Middleton 2019 [50]	Site Clinical Champions from both ED and stroke unit	Registered nurse working in ED and stroke unit	Not reported	Not reported	Name given to champion: site clinical champions Number of champions per site: two (1 ED champion, 1 stroke ward champion) Length of training: No training specifically for site champion. Components of training: N/A Who delivered training / disseminated material / provided support: Nurse researcher who had received training. National opinion leaders were present at first workshop	Site visits every 3 months, teleconferences every 3 months, telephone and email support if initiated by site champion. Specific detail about the content within support 'sessions', or who delivered this support was not reported	No
Mudge, 2022 [40, 41]	Site clinical champion in acute medical ward. Comprised of a competitive recruitment process focused on skills and attributes of facilitation (e.g. self-awareness, communication, interpersonal and assessment skills)	Nurse or Allied health professional	Mid-career (≥ 4 years)	Not reported	Name given to implementation champion: Site facilitator Number of champions per site: One per site Length of training: 4 × half-day initial group training sessions Components of training: didactic and interactive content (based on i-PARIHS facilitation guide), evidence for age-friendly care principles, prevention and management of hospital-associated complications, provision of key readings, and role modelling of facilitation Who delivered training / disseminated material / provided support: Site facilitators were trained and mentored by 2 experienced facilitators who were senior clinician researchers	• Mentoring over monthly half-day face-to-face peer group meetings • Telephone and email support available between meetings, supporting debriefing, reflection on practice and shared learning • Visits from experienced facilitators at each site 3–6 times before and during implementation, meeting key stakeholders and participating in local work group meetings • Project funding provided 24 h/week experienced facilitator support and project management time; and 16 h/week site facilitator plus 20 h/week multi-professional assistant for each implementation ward	Not reported
Puchalski Ritchie 2021 [47]	Not specified. Letters were sent to intervention health centres, asking that TB-focus Lay health workers be sent for training as peer trainers	Lay health workers providing TB care	Not reported	Not reported	Name given to champion: peer trainer Number of champions per site: 1 Length of training: One week Components of training: Peer trainers trained in content (focused on understanding TB disease transmission and treatment, common reasons for non-adherence) and approach to patient education and counselling to support treatment adherence. Training involved a combination of didactic lectures, interactive discussions, and role playing to allow for practice and supportive feedback. The final day of training included an interactive discussion of potential approaches to the cascade training, including options for addressing anticipated challenges to training Received training materials (training manual in Chichewa, stationary, training log book) and a supply of clinical support tools for their site, also in Chichewa Who delivered training / disseminated material / provided support: training by master trainer, provided in English, with support from a sociolinguistic level translator, and a second study team member in attendance to support training in one large district	Peer trainers free to contact the study team by phone with questions or concerns as needed Peer trainers brought together at the end of the cascade training period and then quarterly for the remainder of the implementation period Peer trainers asked to document the number of lay health workers trained; changes/additions made to cascade training; challenges to training or implementation; questions or concerns for discussion with the study team or peer trainer group. Log books were reviewed by the study team at quarterly meetings	Not reported
Resnick 2021 [53]	Not specified. Commented that it was a “facility identified champion”	Not specified	Not reported	No, however site goals were developed	Name given to champion: Champion Number of champions per site: Unclear (although likely to be 1 per site), supported by a stakeholder team Length of training: Formal champion-specific training was unspecified (mode, format, duration), however meetings (2 h, monthly for 12-months) between external facilitator, champion and involved stakeholders. Champion training included: (1) Research Nurse Facilitator teaches the champion to evaluate and match residents’ underlying physical capability and preferences with service plan goals and (2) The Research Nurse Facilitator works with the champion to implement techniques that motivate direct care workers, residents, and families to engage in function-focused care activities Components of training: N/A Who delivered training / disseminated material / provided support: Research Nurse Facilitator	• Initial Stakeholder Team Meeting: included overview of the project; roles and responsibilities of research, champion and stakeholder teams; overview of function-focused care, explanation of participatory implementation and evaluation process, established goals, set regular times for monthly meetings (just intervention group) • Joint assessment with Research Nurse Facilitator of environment and policy assessments, evaluation of patient capabilities and service plans, mentoring and motivating implementation of function-focused care techniques (both control and intervention group)	No
Resnick 2021 [49]	Site Champion; any staff member who expressed a desire to work with the research facilitator and other staff to make a practice change in the residential aged care facility	Not reported	Not reported	Not reported	Name given to champion: Internal champion/site champion Number of champions per site: 1 per site Length of training: Formal training directly to champion was unspecified. However, it is stated that “the champion uses self-efficacy based techniques to motive staff and they’re trained to complete the use of behavioural interventions for BPSD with staff to provide feedback on their care approach” Components of training: Specific components of champion training were unspecified. However in initial training, roles and responsibilities were explained, Who delivered training / disseminated material / provided support: research facilitator	Site champion received coaching from the research facilitator to support staff to assess residents and use behavioural approaches to prevent and manage BPSD	No
Rycroft-Malone 2012 [44]	Opinion leaders were identified by key contacts at the NHS Trusts through a nomination process based on criteria	Not reported	Not reported	Not reported	Name given to implementation champion: Plan-do-study-act facilitator Number of champions per site: 1 champion for group three (*n* = 6) Length of training: 1-day training session for the local champions (group three) was provided. Training on the use of the web-based resource was provided to opinion leaders (group two) at the start of the implementation phase Components of training: Not reported Who delivered training / disseminated material / provided support: Not reported	None	No
Seers 2018 [13]	Experienced staff member nominated by manager to work with external facilitators to implement urinary incontinence recommendation Note: only 6/16 sites recruited internal facilitator who met essential criteria and stayed in role for intervention period	Registered nurse working in residential care	Not reported	Training content presented	Name given to champion: Internal facilitator Number of champions per site: 1 leader, 1 buddy Length of training: Residential programs of 3 days or 5 days (2 intervention arms) Components of training: Educational materials (PowerPoint presentations) provided to intervention and control sites. Face-to-face delivery of education about management of urinary incontinence, education about implementing evidence in local context, guide to knowledge translation, skills training re knowledge translation, support to create action plans to address implementation Who delivered training / disseminated material / provided support: expert facilitators from research team (nurse academics) with > 20 years’ experience each	Intervention group 1: 10 days over 12 months to work locally on the implementation and evaluation of recommendations, supported by 12 half-days for monthly teleconferences and self-directed study (16 days in total). Support via monthly telephone group supervision and email communication Intervention group 2: 20 days to work on the local implementation and evaluation of the recommendations, supported by 24 half-day learning groups via teleconferencing, and 12 half-days for self-directed study (38 days in total)	Yes (additional papers)

**Table 4:** Summary of champion identification, training, support, and practice outcomes in included trials (*n* = 15)

		**Champion Identification**	**Champions per site**	**Champion clinical background**	**Years of clinical experience**	**Training provided (Y/N)**	**Training provider**	**Length of training**	**Follow up support**	**Overall trial effectiveness (practice outcomes)?**
1	Acolet 2011 [48]	Not reported	1	Not reported	Not reported	Yes	Research team	2-day workshop	Email and telephone contact/support for 2–3 months	Improvement in 1 of 3 practice outcomes
2	Ayieko 2011 [42]	Hospital selected	Not reported	Nurse	Not reported	Yes	Research team	5.5-day program	Weekly phone calls, 2–3 monthly site visits and booster training	Improvement in 10/18 practice outcomes
3	Bailey 2021 [45]	Not reported	1–2	Doctor or Nurse	Not reported	Yes	Research team	2-day in-person	Availability as required and bi-weekly conference calls	No change between groups
4	Bunce 2020 [39]	Criteria-informed	1–2	Various	Not reported	Yes	Research team	2-day, F2F training	Monthly emails, webinars and technical support tailored to organisation	No change between groups
5	Cadilhac 2022 [43]	Criteria-informed	1	Various	Not reported	Yes	Research team	Not specified	Telephone or email support over 2 months	Improvement in 4 of 10 practice outcomes
6	Duclos 2022 [51]	Not reported	1	Various	Not reported	Yes	Research team	3 × one-day sessions	No follow-up	Improvement in all outcomes
7	Johnston 2007 [46]	Criteria-informed	Not reported	Nurse	Not reported	Yes	Research team	2-day workshop	As requested	Improvement in 2 of 4 outcomes
8	Llewelyn 2023 [52]	Not reported	1	Microbiologist	Not reported	No	Not reported	Not reported	No	Non-significant improvement in practice outcomes
9	Middleton 2019 [50]	Not reported	1–2	Nurse	Not reported	Yes	Research team	Not specified	3-monthly site visits, Monthly emails, ad-hoc (as needed)	No change
10	Mudge 2022 [40, 41]	Criteria-informed	1	Various	4 + yrs clinical experience	Yes	Research team	X4, half-day training sessions	10–12 group mentoring sessions	No change
11	Puchalski-Ritchie 2021 [47]	Hospital selected	1	Lay health worker	Not reported	Yes	Research team	5-days	Quarterly	No change
12	Resnick 2021 [53]	Not reported	1	Not reported	Not reported	Yes	Research team	Not specified	Yes, 2-h monthly	Improvements in policy and effectiveness outcomes
13	Resnick 2021 [49]	Volunteered	Unclear	Not reported	Not reported	Yes	Research team	Not specified	Yes, monthly	No change
14	Rycroft-Malone 2012 [44]	Criteria-informed	1	Not reported	Not reported	Yes	Not reported	1-day training	None reported	No change
15	Seers 2018 [13]	Criteria-informed	1–2	Nurse	Not reported	Yes	Research team	3- or 5-days training	Regular, monthly	No change

*FU* Follow up post initial training

### Site champion training provided

Specific champion training was provided in 12 (80%) studies [13, 39, 41, 42, 44–49, 51, 53] and was delivered by research team members. Length of training ranged from 1-day to a 5.5-day program. Generally, limited detail was provided about the components of champion training, however from the eleven studies that did report some details [13, 39, 41, 44–48, 51, 53] training focused on topic content e.g. current evidence base and best practice; working with the team e.g. behaviour change principles and champions as leaders; and process related content e.g. use of study-developed resources and action planning for team change. Five studies [41, 46, 47, 51, 53] used champion role-play and role-modelling to practice and refine skills gained during training. See Table 5 for details. No study explicitly stated if site champions identified personal learner goals at the commencement of training, nor stated the champion learning objectives. No studies reported on assessing or micro-credentialing the champions new learning, nor reviewed attainment of champion learning goals.

**Table 5:** Components of training delivered, from the 11 studies that reported the details^a^

	Acolet, 2011 [48]	Bailey, 2021 [45]	Bunce, 2020 [39]	Duclos 2020 [51]	Johnston 2007 [46]	Mudge 2023 [40, 41]	Punchalski Richie, 2021 [47]	Resnick 2021 [53]	Resnick 2021 [49]	Rycroft-Malone, 2012 [44]	Seers, 2018 [13]
Topic Content											
Current practice	✓	✓						✓	✓		✓
Education of best-practice	✓	✓			✓	✓	✓				✓
Benchmarking of practice	✓										
Use of guidelines / toolkit		✓	✓	✓	✓	✓	✓	✓	✓		
Capacity Building Training											
Theory of organisational change	✓										✓
Champions as leaders / roles and responsibilities	✓	✓	✓	✓	✓	✓		✓	✓		
Behaviour change principles	✓										✓
Practical skills to support healthcare staff	✓	✓			✓	✓	✓	✓	✓		✓
Role play for providing feedback				✓	✓	✓	✓	✓			
Approaches to cascade training		✓					✓				
Study-related processes											
Introduction to tools and practice of change	✓	✓	✓	✓	✓	✓				✓	
Tools for practice of change	✓	✓		✓	✓	✓	✓	✓	✓		
Action planning	✓				✓	✓					✓

^a^Ayieko et al. [42] provided 5.5-days training to the site champions, but limited reporting precluded this study from our table

### Training maintenance strategies for champions

Following the initial champion training, 11 studies provided follow up support which varied in mode (email, telephone, in-person), regularity (ad-hoc basis, weekly, monthly, quarterly) and duration (not reported, however one trial reported up to 14-h of ‘booster training’ for select sites [42]). One study reported establishing a peer support network [47], however no other studies reported establishing or providing ongoing community of practice or follow-up post-trial completion.

## Discussion

Understanding how change champions in healthcare are selected and supported to take on their championing role has been until recently, a neglected area of implementation science. Therefore, this rapid systematic review was conducted to identify the education, training, and support provided to individuals in change champion roles within implementation clinical trials. Our review found that one champion per physical site was most common, and champions were usually senior clinical members of staff, selected by the participating healthcare organization rather than the research team. Formal training was provided to most champions, most often delivered by research team members considered experts in implementation. Training duration varied widely. There was a lack of information on training content, but where provided, training focused mostly on topic content and process-related education. No studies reported using an adult learning theory or personalized learning objectives to guide the training and preparation programs.

The absence of personalised learning objectives and subsequently customised training for the designated champions in the included trials suggest that in the implementation RCT paradigm, the role of champion is not recognized as one with potential for individual learning and development.

Although several studies assessed self-reported confidence in own skills, no included trials assessed champions’ knowledge gain. There was no acknowledgment in any studies that training and preparation tasks were underpinned or informed by adult learning theory, nor was enough detail provided for content to be mapped to theory or principles of adult learning. There is likely an implicit assumption that health professionals, especially senior professionals, are equipped to take on a champion role and have the necessary interpersonal and management skills required for successful completion. This assumption is not new; previous research, including a recent systematic review, has consistently identified the lack of learning andragogy in the design of healthcare professional education [55–58]. Qualitative research suggests that experienced clinicians as learners value learning elements consistent with adult learning theory, such as reflective discussions, evaluating performance outcomes, and self-directed learning [59]. Without theory-informed training designed to optimize adult learning outcomes, champions may not respond optimally to the training provided, which can negatively impact the uptake or adoption of information and skills.

Across included trials, there was also a lack of follow up support, regular feedback or behaviour regulation of championing practice – potentially a further limitation in champion preparation and training to date. Adult learners require the opportunity to build on existing knowledge, implement, and reflect which are central to acquiring and refining knowledge and skills [18]. These skills can be acquired through regular contact and mentorship an implementation expert (or research team in the context of this review) who can guide and facilitate this learning in a supportive way [18]. Only one included trial in this review [42] explicitly reported on building role play and modelling training, along with regular follow up, into their program. Applying what we know about the importance of reinforcement to build and retain knowledge in adult learners, even if comprehensive training programs (e.g. two or more days) were offered at the commencement of the trial, this may not be enough to support champions to effect change within the organization. We suggest that it is not possible to separate out training and ongoing support from the effectiveness of the champion package, and may explain the mixed results to date for this implementation intervention.

A recent systematic integrative review of attributes required by an implementation support practitioner reported that adaptability, effective communication skills, and a solid foundation in implementation science are fundamental to successful capacity building in teams, and implementation of change [18]. In areas outside of healthcare, such as early childhood education, micro-credentialing for implementation support practitioners has been reported to be integral for change in practice, with all training and education focused on evidence-based coaching practices, using data for decision-making, reflective practice, and practical application of theory [60]. Our review found that most champion preparation training focused on topic content education (i.e. best-practice recommendations), and study-related processes (such as use of study-developed tools to support the championing role). Only two trials providing education on behaviour change principles [13, 48]. In their process evaluation of a large implementation trial, Bunce and colleagues [39] found in sites that achieved practice change (*n* = 5) champions were supported by ‘emergent champions’, someone internal to provide additional support to the champion and champion activities. Similarly, in Ayieko et al.’s [42] trial, the addition of booster training for champions, in conjunction with cascade training, seemed to mitigate the impact of staff turnover, ultimately producing more than only one champion per site.

While evidence of effectiveness had already shown that site champions can be a useful implementation intervention [8, 15, 16, 37], our review suggests that this effectiveness may be associated with the pre-existing skills of the individual champion, and may not be due to solely training and support provided. Given the lack of structured training in most trials, the effectiveness or influence a champion may have is very likely affected by the individual in the position, the skills and experiences they bring to the role, how they relate to the others, as well as contextual and temporal factors at the site [51]. Some of these skills may be further developed or refined by training and appropriate support, however, without baseline information on *who* are in these positions (qualifications, professional background, years of experience), the attributes they possess (interpersonal skills, confidence to provide feedback to staff, social standing within their team), how well they are being prepared (i.e. content of training) and supported in the role (frequency, duration, mode of mentorship), there will remain many unknowns about the “champion” as an implementation intervention. Further, it is unclear whether champions supporting implementation of different evidence-based interventions (e.g. introducing a new evidence-based practice versus improving performance in a familiar practice, use of technology versus a person-facing intervention) require different training, education and support. Until clear, consistent, and detailed reporting is made available, we will not be further advanced in knowing *how best* the champion strategy can work, how we best build capacity for people in such roles, how training has been or should be personalised or tailored to the champion, nor can it be replicated and produced at scale.

Taken together, aligning information from this review with adult learning theories, we suggest that: i) a larger portion of champion training and preparation tasks should be dedicated to principles of implementation science, team capacity building and interpersonal skills rather than content-specific education (i.e. evidence-based practice); ii) champion learning goals should be set at the commencement of training, and training tailored or personalised; iii) training should be informed and underpinned by adult learning principles; inclusive of self-paced learning, modules delivered in small doses, and practical skills taught via role modelling and reflective practice; iv) knowledge checks, assessments or micro-credentialing of new skills should be conducted to ensure competencies gained and acknowledge new learning; and, v) regular and interactive sessions with mentors / supports should be provided, to foster ongoing champion support.

A strength of this review is that available details on the champions have been synthesized across studies. The study employed a rapid review method, which, while efficient and every effort was made to include relevant studies, may have inadvertently not identified all eligible trials. Detailed information about champion training was often not reported in the published trials and while efforts were made to obtain further details from separate process evaluation papers and by contacting corresponding authors, detailed information was seldom provided. Additionally, the supplementary files of Ayieko et al.’s [42] study and Duclos’s [51] study were in a language not accessible to our research team, which restricted our ability to include this data in our analysis. These factors should be considered when interpreting the findings of this review, as they may affect the comprehensiveness of the presented information.

## Conclusion

Change champions in implementation trials usually received some degree of education and training, however the content of the education/training package was reported in very limited detail. Education of champions, when provided, did not align with adult learning principles – no studies reported personal baseline characteristics or learning goals of champions, nor champion knowledge outcomes. Follow up support for champions was found to be minimal in most trials, and when follow up was reported, no detail was provided about the *type or content* of support provided in these sessions. Future trials should address these findings, by structuring education and training to optimize adult learning, and by providing detailed accounts of the education and training processes and content in publications to enhance transparency and replicability. This approach would contribute to the refining of existing methodologies and prevent the need for designing new champion training and preparation for each trial. By doing so, we can ensure a more systematic and theory-driven approach to champion training in implementation trials, potentially enhancing their effectiveness and impact.

## Supplementary Information

Supplementary Material 1.

## Data Availability

All data generated or analysed during this study are included in this published article [and its supplementary information files].

## Abbreviations

cRCT: Cluster Randomised Controlled trial
RCT: Randomised Control Trial
PROSPERO: International Prospective Register of Systematic reviews
TIDieR: Template for Intervention Description and Replication Framework

## References

[1] Jolliffe L, Hoffmann T, Laver K, et al. Stroke rehabilitation research translation in Australia: a survey of clinical trialists. Disabil Rehabil. 2022;44(10):2131–7. 32847418 10.1080/09638288.2020.1807619

[2] Lenfant C. Shattuck lecture–clinical research to clinical practice–lost in translation? N Engl J Med. 2003;349(9):868–74. 12944573 10.1056/NEJMsa035507

[3] Grol R, Wensing M, Eccles M, et al. Improving patient care: the implementation of change in health care. John Wiley & Sons; 2013.

[4] Katz J, Wandersman A. Technical assistance to enhance prevention capacity: a research synthesis of the evidence base. Prev Sci. 2016;17(4):417–28. 26858179 10.1007/s11121-016-0636-5 PMC4839040

[5] Leeman J, Calancie L, Hartman MA, et al. What strategies are used to build practitioners’ capacity to implement community-based interventions and are they effective?: a systematic review. Implement Sci. 2015;10(1):1–15. 26018220 10.1186/s13012-015-0272-7 PMC4449971

[6] Powell BJ, Waltz TJ, Chinman MJ, et al. A refined compilation of implementation strategies: results from the Expert Recommendations for Implementing Change (ERIC) project. Implementat Sci. 2015;10(1):21. 10.1186/s13012-015-0209-1 PMC4328074 25889199

[7] Michie SAL, West R. The behaviour change wheel: a guide to designing interventions. UK: Silverback Publishing; 2014.

[8] Cranley LA, Cummings GG, Profetto-McGrath J, et al. Facilitation roles and characteristics associated with research use by healthcare professionals: a scoping review. BMJ Open. 2017;7(8):e014384. 28801388 10.1136/bmjopen-2016-014384 PMC5724142

[9] Greenhalgh T, Robert G, Macfarlane F, et al. Diffusion of innovations in service organizations: systematic review and recommendations. Milbank Q. 2004;82(4):581–629. 15595944 10.1111/j.0887-378X.2004.00325.x PMC2690184

[10] Albers B, Metz A, Burke K. Implementation support practitioners – a proposal for consolidating a diverse evidence base. BMC Health Serv Res. 2020;20(1):368. 10.1186/s12913-020-05145-1 PMC7193379 32357877

[11] Bührmann L, Driessen P, Metz A, et al. Knowledge and attitudes of Implementation Support Practitioners—Findings from a systematic integrative review. PLoS ONE. 2022;17(5):e0267533. 35544529 10.1371/journal.pone.0267533 PMC9094539

[12] Miech EJ, Rattray NA, Flanagan ME, et al. Inside help: An integrative review of champions in healthcare-related implementation. SAGE Open Med. 2018;6:2050312118773261. 29796266 10.1177/2050312118773261 PMC5960847

[13] Seers K, Rycroft-Malone J, Cox K, et al. Facilitating Implementation of Research Evidence (FIRE): an international cluster randomised controlled trial to evaluate two models of facilitation informed by the Promoting Action on Research Implementation in Health Services (PARIHS) framework. Implement Sci. 2018;13(1):137. 10.1186/s13012-018-0831-9 PMC6238407 30442174

[14] Wang A, Pollack T, Kadziel LA, et al. Impact of practice facilitation in primary care on chronic disease care processes and outcomes: a systematic review. J Gen Intern Med. 2018;33(11):1968–77. 30066117 10.1007/s11606-018-4581-9 PMC6206351

[15] Bornbaum CC, Kornas K, Peirson L, et al. Exploring the function and effectiveness of knowledge brokers as facilitators of knowledge translation in health-related settings: a systematic review and thematic analysis. Implement Sci. 2015;10(1):1–12. 26589972 10.1186/s13012-015-0351-9 PMC4653833

[16] Santos WJ, Graham ID, Lalonde M, et al. The effectiveness of champions in implementing innovations in health care: a systematic review. Implement Sci Commun. 2022;3(1):80. 10.1186/s43058-022-00315-0 PMC9308185 35869516

[17] Albers B, Metz A, Burke K, et al. Implementation Support Skills: Findings From a Systematic Integrative Review. Res Soc Work Pract. 2021;31(2):147–70.

[18] Metz A, Albers B, Burke K, et al. Implementation Practice in Human Service Systems: Understanding the Principles and Competencies of Professionals Who Support Implementation. Hum Serv Organ Manag Leadership Govern. 2021;45(3):238–259.

[19] Morena AL, Gaias LM, Larkin C. Understanding the Role of Clinical Champions and Their Impact on Clinician Behavior Change: The Need for Causal Pathway Mechanisms [Hypothesis and Theory]. Front Health Serv. 2022;2. 10.3389/frhs.2022.896885 PMC10012807 36925794

[20] Bonawitz K, Wetmore M, Heisler M, et al. Champions in context: which attributes matter for change efforts in healthcare? Implement Sci. 2020;15:1–10. 32762726 10.1186/s13012-020-01024-9 PMC7409681

[21] Stander J, Grimmer K, Brink Y. Training programmes to improve evidence uptake and utilisation by physiotherapists: a systematic scoping review. BMC Med Educ. 2018;18(1):1–12. 29334943 10.1186/s12909-018-1121-6 PMC5769325

[22] Mosson R, Augustsson H, Bäck A, et al. Building implementation capacity (BIC): a longitudinal mixed methods evaluation of a team intervention. BMC Health Serv Res. 2019;19(1):1–12. 31064362 10.1186/s12913-019-4086-1 PMC6505288

[23] Park JS, Moore JE, Sayal R, et al. Evaluation of the “Foundations in Knowledge Translation” training initiative: preparing end users to practice KT. Implement Sci. 2018;13(1):1–13. 29695267 10.1186/s13012-018-0755-4 PMC5918493

[24] Provvidenza C, Townley A, Wincentak J, et al. Building knowledge translation competency in a community-based hospital: a practice-informed curriculum for healthcare providers, researchers, and leadership. Implement Sci. 2020;15(1):1–12. 32620129 10.1186/s13012-020-01013-y PMC7333339

[25] Moore JE, Rashid S, Park JS, et al. Longitudinal evaluation of a course to build core competencies in implementation practice. Implement Sci. 2018;13(1):1–13. 30081921 10.1186/s13012-018-0800-3 PMC6080520

[26] Tricco AC, Antony J, Zarin W, et al. A scoping review of rapid review methods. BMC Med. 2015;13(1):224. 10.1186/s12916-015-0465-6 PMC4574114 26377409

[27] Klerings I, Robalino S, Booth A, et al. Rapid reviews methods series: Guidance on literature search. BMJ Evidence-Based Medicine. 2023;28(6):412–7. 37076268 10.1136/bmjebm-2022-112079 PMC10715472

[28] Garritty C, Gartlehner G, Nussbaumer-Streit B, et al. Cochrane Rapid Reviews Methods Group offers evidence-informed guidance to conduct rapid reviews. J Clin Epidemiol. 2021;130:13–22. 33068715 10.1016/j.jclinepi.2020.10.007 PMC7557165

[29] Nussbaumer-Streit B, Sommer I, Hamel C, et al. Rapid reviews methods series: Guidance on team considerations, study selection, data extraction and risk of bias assessment. BMJ Evid Based Med. 2023;28(6):418–23. 37076266 10.1136/bmjebm-2022-112185 PMC10715469

[30] Maher CG, Sherrington C, Herbert RD, et al. Reliability of the PEDro scale for rating quality of randomized controlled trials. Phys Ther. 2003;83(8):713–21. 12882612

[31] de Morton NA. The PEDro scale is a valid measure of the methodological quality of clinical trials: a demographic study. Aust J Physiother. 2009;55(2):129–33. 19463084 10.1016/s0004-9514(09)70043-1

[32] Elkins MR, Moseley AM, Sherrington C, et al. Growth in the Physiotherapy Evidence Database (PEDro) and use of the PEDro scale. Br J Sports Med. 2013;47(4):188–9. 23134761 10.1136/bjsports-2012-091804

[33] Maher C, Sherrington C, Herbert R, et al. Reliability of the PEDro scale for rating methodological quality of randomized controlled trials. Phys Ther. 2003;83:713–21. 12882612

[34] Hoffmann TC, Glasziou PP, Boutron I, et al. Better reporting of interventions: template for intervention description and replication (TIDieR) checklist and guide. BMJ : British Medical Journal. 2014;348:g1687. 24609605 10.1136/bmj.g1687

[35] Popay J, Roberts H, Sowden A, et al. Guidance on the Conduct of Narrative Synthesis in Systematic Reviews: a product from the ESRC Methods Programme. Lancaster, UK: University of Lancaster; 2006.

[36] Lisy K, Porritt K. Narrative Synthesis: Considerations and challenges. JBI Evid Implement. 2016;14(4).

[37] Moussa L, Garcia-Cardenas V, Benrimoj SI. Change Facilitation Strategies Used in the Implementation of Innovations in Healthcare Practice: A Systematic Review. J Change Manag. 2019;19(4):283–301.

[38] Page MJ, McKenzie JE, Bossuyt PM, et al. The PRISMA 2020 statement: an updated guideline for reporting systematic reviews. BMJ. 2021;372:n71. 33782057 10.1136/bmj.n71 PMC8005924

[39] Bunce AE, Gruß I, Davis JV, et al. Lessons learned about the effective operationalization of champions as an implementation strategy: results from a qualitative process evaluation of a pragmatic trial. Implement Sci. 2020;15(1):87. 10.1186/s13012-020-01048-1 PMC7528604 32998750

[40] Mudge AM, McRae P, Banks M, et al. Effect of a Ward-Based Program on Hospital-Associated Complications and Length of Stay for Older Inpatients: The Cluster Randomized CHERISH Trial. JAMA Intern Med. 2022;182(3):274–82. 35006265 10.1001/jamainternmed.2021.7556 PMC8749692

[41] Mudge AM, McRae P, Young A, et al. Implementing a ward-based programme to improve care for older inpatients: process evaluation of the cluster randomised CHERISH trial. BMC Health Serv Res. 2023;23(1):668. 10.1186/s12913-023-09659-2 PMC10283300 37344776

[42] Ayieko P, Ntoburi S, Wagai J, et al. A Multifaceted Intervention to Implement Guidelines and Improve Admission Paediatric Care in Kenyan District Hospitals: A Cluster Randomised Trial. PLoS Med. 2011;8(4):e1001018. 21483712 10.1371/journal.pmed.1001018 PMC3071366

[43] Cadilhac DA, Marion V, Andrew NE, et al. A Stepped-Wedge Cluster-Randomized Trial to Improve Adherence to Evidence-Based Practices for Acute Stroke Management. Jt Comm J Qual Patient Saf. 2022;48(12):653–64. 36307360 10.1016/j.jcjq.2022.09.003

[44] Rycroft-Malone J, Seers K, Crichton N, et al. A pragmatic cluster randomised trial evaluating three implementation interventions. Implement Sci. 2012;7(1):80. 10.1186/1748-5908-7-80 PMC3457838 22935241

[45] Bailey FA, Williams BR, Goode PS, et al. Comparison of two methods for implementing comfort care order sets in the inpatient setting: a cluster randomized trial. J Gen Intern Med. 2021;36(7):1928–36. 33547573 10.1007/s11606-020-06482-x PMC8298677

[46] Johnston CC, Gagnon A, Rennick J, et al. One-on-one coaching to improve pain assessment and management practices of pediatric nurses. J Pediatr Nurs. 2007;22(6):467–78. 18036467 10.1016/j.pedn.2007.07.004

[47] Puchalski Ritchie LM, Kip EC, Mundeva H, et al. Process evaluation of an implementation strategy to support uptake of a tuberculosis treatment adherence intervention to improve TB care and outcomes in Malawi. BMJ Open. 2021;11(7):e048499. 34215610 10.1136/bmjopen-2020-048499 PMC8256754

[48] Acolet D, Allen E, Houston R, et al. Improvement in neonatal intensive care unit care: a cluster randomised controlled trial of active dissemination of information. Arch Dis Child Fetal Neonatal Ed. 2011;96(6):F434–9. 21393310 10.1136/adc.2010.207522

[49] Resnick B, Van Haitsma K, Kolanowski A, et al. Implementation of the Evidence Integration Triangle for behavioral and psychological symptoms of dementia (EIT-4-BPSD) in care communities. Nurs Outlook. 2021;69(6):1058–1071. 10.1016/j.outlook.2021.06.004 PMC8678150 34332762

[50] Middleton S, Dale S, Cheung NW, et al. Nurse-Initiated Acute Stroke Care in Emergency Departments. Stroke. 2019;50(6):1346–55. 31092163 10.1161/STROKEAHA.118.020701

[51] Duclos A, Chollet F, Pascal L, et al. Effect of monitoring surgical outcomes using control charts to reduce major adverse events in patients: cluster randomised trial. BMJ. 2020;371:m3840. 33148601 10.1136/bmj.m3840 PMC7610189

[52] Llewelyn MJ, Budgell EP, Laskawiec-Szkonter M, et al. Antibiotic review kit for hospitals (ARK-Hospital): a stepped-wedge cluster-randomised controlled trial. Lancet Infect Dis. 2023;23(2):207–21. 36206793 10.1016/S1473-3099(22)00508-4

[53] Resnick B, Boltz M, Galik E, et al. Testing the Implementation of Function-focused Care in Assisted Living Settings. J Am Med Dir Assoc. 2021;22(8):1706-1713.e1. 33132018 10.1016/j.jamda.2020.09.026 PMC8081737

[54] Cashin AG, McAuley JH. Clinimetrics: Physiotherapy Evidence Database (PEDro) Scale. J Physiother. 2020;66(1):59. 31521549 10.1016/j.jphys.2019.08.005

[55] Benner PE, Tanner CA, Chesla CA. Expertise in nursing practice: Caring, clinical judgment, and ethics. Springer Publishing Company; 2009.

[56] Waterfield J. Two approaches to vocational education and training. A view from pharmacy education. J Vocat Educ Train. 2011;63(2):235–246.

[57] Waterfield J. Using Bourdieu’s theoretical framework to examine how the pharmacy educator views pharmacy knowledge. Am J Pharm Educ. 2015;79(10). 10.5688/ajpe7910153 PMC4749901 26889065

[58] Mulder M, Roelofs E. A Critical Review of Vocational Education and Training Research and Suggestions for the Research Agenda. 2012.

[59] Spies C, Seale I, Botma Y. Adult learning: What nurse educators need to know about mature students. Curationis. 2015;38(2):1494. 26842085 10.4102/curationis.v38i2.1494 PMC6091729

[60] Ward C, Ihlo T, Ryan Jackson K, et al. Effective implementation capacity to impact change within state education systems to support students with disabilities. J Disabil Policy Stud. 2023;34(2):104–14.

