# Supplementary material for: Training and education provided to local change champions within implementation trials: a rapid systematic review
Source: Implement Sci. 2025 Feb 5;20:8. doi: 10.1186/s13012-025-01416-9 (PMC11796244; doi:10.1186/s13012-025-01416-9)
Supplement: Supplementary file 1 — Supplementary Material 1. [file 13012_2025_1416_MOESM1_ESM.docx]

**Supplementary File 1:** Search strategy for Part A; systematic reviews and/or scoping reviews that addressed the effectiveness of and/or important attributes for: *champions, knowledge brokers*, *facilitators*, and *implementation support practitioners.*

Conducted in Scopus on 1^st^ November 2022, and again in 22^nd^ November 2023.

**Rapid Review: Scopus Search Strategy**

| 1. (coach* OR facilit* OR champion* OR “knowledge broker*” OR “implementation lead*” OR “implementation support*” OR leader) | 2,683, 989 |
| --- | --- |
| 1. ("evidence based" OR "evidence-based") OR (("evidence based" OR "evidence-based") W/2 (practice OR guidelines OR recommendations)) OR ("clinical practice guidelines") OR (improvement W/2 (quality OR service OR performance OR practice)) | 703,210 |
| 1. (implementation W/2 (support OR strateg* OR practitioner OR research)) OR (implementation) OR (research W/2 (adoption OR translation)) | 1,788,762 |
| 1. 2 OR 3 | 2, 423, 657 |
| 1. (strateg* W/2 (facilitat* OR design)) OR (strateg*) OR (change W/2 (management OR facilitat*)) | 4,201,442 |
| 1. ("health" W/2 (care OR healthcare)) OR ("healthcare" W/2 (organisations OR management OR personnel OR professional)) OR ("health AND care" W/2 (organisations OR management OR personnel OR professional)) OR (health W/2 (research OR analysis)) | 2,255, 368 |
| 1. (review W/2 (systematic OR scoping OR rapid OR Cochrane)) OR (data W/2 (synthesi* OR extract*)) | 700,310 |
| 1. 1 AND 4 AND 5 AND 6 AND 7 | 1,395 |
| 1. DATE RANGE 2015 to 2022 | 937 |

**Supplementary File 2:** Implementation Experts contacted via email

| Name | Position |
| --- | --- |
| Professor Alison Hutchinson | **Chair in Nursing**  Monash Health, Centre for Nursing Research, Melbourne, Australia  **Professor of Nursing**  Deakin University, School of Nursing and Midwifery, Melbourne, Australia |
| Professor Paul Wilson | **Professor**  The University of Manchester |
| Professor Gregory Aarons | **Professor**  Department of Psychiatry at the University of California, San Diego  **Co-Director**  UC San Diego Dissemination and Implementation Science Center |
| Professor Jeremy Grimshaw | **Senior Scientist, Methodological and Implementation Research**  Ottawa Hospital Research Institute  **Professor, Department of Medicine**  University of Ottawa |
| Professor Allison Metz | **Associate Director, National Implementation Research Network**  University of North Carolina, Frank Porter Graham Development Institute |
| Dr Arwen Bunce | **Academic Researcher**  Oregon Health & Science University |
| Professor Alison Mudge | **Clinical Director of Education and Research**  Department of Internal Medicine and Aged Care at the Royal Brisbane and Women's Hospital |

**Supplementary File 3:** Search strategy for Part B search; to identify trials published after the searches from the included systematic reviews were conducted. Conducted on 14^th^ November 2023.

Santos 2022 Search Strategy completed between 2010 and April 2020. Replicated by our research team, date limited from 01/01/2020 to 14/11/2023 to prevent duplication of findings.

**1. MEDLINE (via OVID)**

| Population: Champions | 1. (champion*).mp. | 10,183 |
| --- | --- | --- |
|  | 1. peer influence/ | 811 |
|  | 1. leadership/ | 48,034 |
|  | 1. peer group/ | 24,302 |
|  | 1. 3 AND 4 | 383 |
|  | 1. 1 OR 2 OR 5 | 11,372 |
| Concept: Knowledge translation/evidence base practice | 1. (ebp or ebm or ebn or cpg* or best practice* or (evidence adj2 practice*)).mp. | 124,472 |
|  | 1. (guideline* adj2 (implement* or adher*)).mp. | 44,166 |
|  | 1. (quality adj1 (improv* or manag*)).mp. | 126,012 |
|  | 1. (research adj2 (‘use’ or utili* or adopt* or implement* or disseminat* or uptake or transfer* or translat* or support)).mp. | 10,435,402 |
|  | 1. (knowledge adj2 (‘use’ or utili* or adopt* or implement* or disseminat* or uptake or transfer* or translat* or support)).mp. | 24,118 |
|  | 1. (evidence adj2 (‘use’ or utili* or adopt* or implement* or disseminat* or uptake or transfer* or translat* or support)).mp. | 63,583 |
|  | 1. ((innovation adj2 (adopt* or diffus*)) or (implementation adj2 (science* or research)) or (innovation or diffusion)).mp | 370,276 |
|  | 1. exp Evidence-Based Practice/ or Information Dissemination/ | 112,076 |
|  | 1. guideline adherence/ or quality assurance, health care/ or quality improvement/ or Outcome Assessment, Health Care/ or Process Assessment, Health Care/ or "Outcome and Process Assessment, Health Care"/ or Safety Management/ | 247,429 |
|  | 1. "diffusion of innovation"/ or implementation science/ or technology transfer/ or Organizational Innovation/ | 45,441 |
|  | 1. OR/7-16 | 11,079,791 |
| Context: Healthcare | 1. (health adj2 care or healthcare).mp. | 1,231,323 |
|  | 1. ((health care adj2 (professional* or personnel* or provider* or occupation*)) or (healthcare adj2 (professional* or personnel* or provider* or occupation*)) or nurse* or physician* or dentist* or (allied adj2 profession*)).mp. | 1,278,962 |
|  | 1. Patient care/ or "Delivery of Health Care"/ or Health Services Administration/ or Quality of Health Care/ or health resources/ or "health services needs and demand"/ or Health Services/ or Health Services Research/ or Hospitals/ or Ambulatory Care Facilities/ | 428,668 |
|  | 1. Health Personnel/ or Health Occupations/ or practice patterns, dentists'/ or practice patterns, nurses'/ or practice patterns, physicians'/ or professional practice gaps/ or "attitude of health personnel"/ or Interprofessional Relations/ or Health Knowledge, Attitudes, Practice/ | 412,858 |
|  | 1. OR/18-21 | 2,439,298 |
| Combinations | 1. 6 AND 17 AND 22 | 2,081 |
| Reduced Timeframe 2020 – Current | | 704 |

**2. PsycINFO (via OVID)**

| 1. champion*.mp | 4,610 |
| --- | --- |
| 1. interpersonal influences/ | 6,259 |
| 1. Leadership/ | 44,860 |
| 1. exp peers/ | 17,671 |
| 1. 3 AND 4 | 262 |
| 1. 1 OR 2 OR 5 | 11,115 |
| 1. (ebp or ebm or ebn or cpg* or best practice* or (evidence adj2 practice*)).mp. | 52,847 |
| 1. (guideline* adj2 (implement* or adher*)).mp. | 4,062 |
| 1. (quality adj1 (improv* or manag*)).mp. | 16,587 |
| 1. (research adj2 ('use' or utili* or adopt* or implement* or disseminat* or uptake or transfer* or translat* or support)).mp | 30,935 |
| 1. (knowledge adj2 ('use' or utili* or adopt* or implement* or disseminat* or uptake or transfer* or translat* or support)).mp. | 16,413 |
| 1. (evidence adj2 ('use' or utili* or adopt* or implement* or disseminat* or uptake or transfer* or translat* or support)).mp. | 22,142 |
| 1. ((innovation adj2 (adopt* or diffus*)) or (implementation adj2 (science* or research)) or (innovation or diffusion)).mp. | 61,855 |
| 1. Evidence Based Practice/ or Information Dissemination/ | 23,247 |
| 1. treatment guidelines/ or quality control/ or "treatment process and outcome measures"/ or patient safety/ or Program Evaluation/ | 29,402 |
| 1. innovation/ | 18,204 |
| 1. OR/7-16 | 210,096 |
| 1. ((health adj2 care) or healthcare).mp. | 293,075 |
| 1. ((health care adj2 (professional* or personnel* or provider* or occupation*)) or (healthcare adj2 (professional* or personnel* or provider* or occupation*)) or nurse* or physician* or dentist* or (allied adj2 profession*)).mp. | 203,126 |
| 1. Health Care Delivery/ or Health Care Services/ or Health Care Administration/ or Health Care Administration/ or Hospitals/ or Outpatient Treatment/ | 94,338 |
| 1. Health Personnel Attitudes/ or Health Personnel/ | 44,248 |
| 1. or/18-21 | 435,716 |
| 1. 6 and 17 and 22 | 320 |
| 1. Reduced Timeframe 2020 – Current | 95 |

**3. CINAHL (EBSCO)**

| 1. (champion*) |  |
| --- | --- |
| 1. (MH "Peer Pressure") |  |
| 1. (MH "Leadership") | 49,817 |
| 1. (MH "Peer Group") | 16,544 |
| 1. S3 AND S4 | 321 |
| 1. S1 OR S2 OR S5 | 9,109 |
| 1. (ebp or ebm or ebn or cpg* or best practice* or (evidence n2 practice*)) | 123,736 |
| 1. (guideline* n2 (implement* or adher*)) | 24,685 |
| 1. (quality n1 (improv* or manag*)) | 126,711 |
| 1. (research n2 (use or utili* or adopt* or implement* or disseminat* or uptake or transfer* or translat* or support)) | 49,977 |
| 1. (knowledge n2 (use or utili* or adopt* or implement* or disseminat* or uptake or transfer* or translat* or support)) | 16,373 |
| 1. (evidence n2 (use or utili* or adopt* or implement* or disseminat* or uptake or transfer* or translat* or support)) | 45,765 |
| 1. ((innovation n2 (adopt* or diffus*)) or (implementation n2 (science* or research)) or (innovation or diffusion) | 74,986 |
| 1. (MH "Evidence-Based Dental Practice") OR (MH "Medical Practice, Evidence-Based") OR (MH "Nursing Practice, Evidence-Based") OR (MH "Professional Practice, Evidence-Based") OR (MH "Occupational Therapy Practice, Evidence-Based") OR (MH "Physical Therapy Practice, Evidence-Based") OR (MH "Professional Practice, Evidence-Based") OR (MH "Professional Practice, Research-Based") OR (MH "Information Management") | 90,290 |
| 1. ((MH "Quality Assurance") OR (MH "Quality Assessment") OR (MH "Quality Improvement")) OR ((MH "Guideline Adherence") OR (MH "Process Assessment (Health Care)") OR (MH "Outcome Assessment")) | 165,475 |
| 1. (MH "Diffusion of Innovation") OR (MH "Implementation Science") | 19,698 |
| 1. S7 OR S8 OR S9 OR S10 OR S11 OR S12 OR S13 OR S14 OR S15 OR S16 | 488,371 |
| 1. (healthcare OR health n2 care) | 790,064 |
| 1. (((health care n2 (professional* or personnel* or provider* or occupation*)) OR ((healthcare n2 (professional* or personnel* or provider* or occupation*)) OR ( nurse* or physician* or dentist* or (allied n2 profession*)) | 925,218 |
| 1. (MH "Patient Care") OR (MH "Health Care Delivery") OR (MH "Health Care Delivery, Integrated") OR (MH "Health Services Administration") OR (MH "Health Care Supervision (Omaha)") OR (MH "Health Services Needs and Demand") OR (MH "Health Services") OR (MH "Quality of Health Care") OR (MH "Health Services Research") OR (MH "Hospitals") OR (MH "Ambulatory Care") | 312,852 |
| 1. (MH "Health Personnel") OR (MH "Health Occupations") OR (MH "Attitude of Health Personnel") OR (MH "Practice Patterns") OR (MH "Interprofessional Relations") | 141,585 |
| 1. S18 OR S19 OR S20 OR S21 | 1,607,880 |
| 1. Timeframe between 2020 – 2023 | 430 |

**5. Nursing and Allied Health (ProQuest)**

| 1. noft((champion*)) | 11,542 |
| --- | --- |
| 1. mainsubject.Exact("peer influence") | 96 |
| 1. mainsubject.Exact("leadership") | 37,086 |
| 1. mainsubject.Exact("peer group") | 1,706 |
| 1. S3 AND S4 | 34 |
| 1. S1 OR S2 OR S5 | 11,671 |
| 1. noft((ebp or ebm or ebn or cpg* or best practice* or (evidence Near/2 practice*))) | 112,748 |
| 1. noft((guideline* Near/2 (implement* or adher*))) | 8,633 |
| 1. noft((quality Near/1 (improv* or manag*))) |  |
| 1. noft((research Near/2 (use or utili* or adopt* or implement* or disseminat* or uptake or transfer* or translat* or support))) | 57,500 |
| 1. noft((knowledge Near/2 (use or utili* or adopt* or implement* or disseminat* or uptake or transfer* or translat* or support))) | 11,051 |
| 1. noft((evidence Near/2 (use or utili* or adopt* or implement* or disseminat* or uptake or transfer* or translat* or support))) | 28,563 |
| 1. noft(((innovation Near/2 (adopt* or diffus*)) or (implementation Near/2 (science* or research)) or (innovation or diffusion))) | 89,540 |
| 1. mainsubject.Exact("evidence-based practice" OR "evidence-based nursing" OR "information dissemination" OR "evidence based practice" OR "evidence based" OR "evidence-based medicine") | 36,645 |
| 1. mainsubject.Exact("process assessment (health care)" OR "safety management" OR "outcome & process assessment (health care)" OR "guideline adherence" OR "quality improvement" OR "outcome assessment (health care)" OR "quality assurance, health care") | 29,898 |
| 1. mainsubject.Exact("organizational innovation" OR "implementation" OR "diffusion of innovation") | 7,109 |
| 1. S7 OR S8 OR S9 OR S10 OR S11 OR S12 OR S13 OR S14 OR S15 OR S16 | 369,948 |
| 1. noft((healthcare OR health Near/2 care)) | 819,133 |
| 1. noft(((health care Near/2 (professional* or personnel* or provider* or occupation*)) ) OR ( (healthcare Near/2 (professional* or personnel* or provider* or occupation*)) ) OR ( nurse* or physician* or dentist* or (allied Near/2 profession*))) | 734,347 |
| 1. mainsubject.Exact("health services research" OR "hospitals" OR "quality of health care" OR "ambulatory care facilities" OR "health resources" OR "health services needs & demand" OR "health services administration" OR "health services" OR "patient care" OR "delivery of health care") | 290,245 |
| 1. mainsubject.Exact("health knowledge, attitudes, practice" OR "health occupations" OR "attitude of health personnel" OR "interprofessional relations" OR "health personnel") | 25,479 |
| 1. S18 OR S19 OR S20 OR S21 | 1,406,230 |
| 1. S6 AND S17 AND S22 | 1,075 |
| 1. Timeframe between 2020 – 2023 | 380 |

**6. Dissertations and Theses Global (ProQuest)**

| 1. noft(champion*) | 6,674 |
| --- | --- |
| 1. noft((ebp or ebm or ebn or cpg* or best practice* or (evidence Near/2 practice*))) | 118,069 |
| 1. noft((guideline* Near/2 (implement* OR adher*))) | 2,209 |
| 1. noft((quality Near/1 (improv* OR manag*))) | 45,316 |
| 1. noft((research Near/2 (use OR utili* OR adopt* OR implement* OR disseminat* OR uptake OR transfer* OR translat* OR support))) | 73,325 |
| 1. noft((knowledge Near/2 (use OR utili* OR adopt* OR implement* OR disseminat* OR uptake OR transfer* OR translat* OR support))) | 21,694 |
| 1. noft((evidence Near/2 (use OR utili* OR adopt* OR implement* OR disseminat* OR uptake OR transfer* OR translat* OR support))) | 23,542 |
| 1. noft(((innovation Near/2 (adopt* OR diffus*)) OR (implementation Near/2 (science* OR research)) OR (innovation OR diffusion))) | 161,405 |
| 1. S2 OR S3 OR S4 OR S5 OR S6 OR S7 OR S8 | 404,906 |
| 1. noft((healthcare OR health Near/2 care)) | 102,993 |
| 1. noft(((health care Near/2 (professional* or personnel* or provider* or occupation*)) ) OR ( (healthcare Near/2 (professional* or personnel* or provider* or occupation*)) ) OR ( nurse* or physician* or dentist* or (allied Near/2 profession*))) | 103,776 |
| 1. S10 OR S11 | 167,459 |
| 1. S1 AND S9 AND S12 | 156 |
| 1. Timeframe between 2020 – 2023 | 38 |

**7. SCOPUS**

| 1. TITLE-ABS-KEY ((champion*)) | 33,656 |
| --- | --- |
| 1. TITLE-ABS-KEY (((ebp OR ebm OR ebn OR cpg* OR “best practice*” OR (evidence W/2 practice* )))) | 296,107 |
| 1. TITLE-ABS-KEY (((guideline* W/2 (implement* OR adher*)))) | 55,919 |
| 1. TITLE-ABS-KEY (((quality W/1 (improv* OR manag*)))) | 520,894 |
| 1. TITLE-ABS-KEY (((research W/2 (use OR utili* OR adopt* OR implement* OR disseminat* OR uptake OR transfer* OR translat* OR support)))) | 213,715 |
| 1. TITLE-ABS-KEY (((knowledge W/2 (use OR utili* OR adopt* OR implement* OR disseminat* OR uptake OR transfer* OR translat* OR support)))) | 100,900 |
| 1. TITLE-ABS-KEY (((evidence W/2 (use OR utili* OR adopt* OR implement* OR disseminat* OR uptake OR transfer* OR translat* OR support )))) | 148,069 |
| 1. TITLE-ABS-KEY ((((innovation W/2 (adopt* OR diffus*)) OR (implementation W/2 (science* OR research )) OR ( innovation OR diffusion )))) | 1,762,484 |
| 1. #2 OR # 3 OR #4 OR #5 OR #6 OR #7 OR #8 | 2,957,950 |
| 1. TITLE-ABS-KEY (((healthcare OR health W/2 care))) | 2,114,889 |
| 1. TITLE-ABS-KEY (((“health AND care” W/2 (professional* OR personnel* OR provider* OR occupation*)) OR (healthcare W/2 (professional* OR personnel* OR provider* OR occupation*)) OR nurse* OR physician* OR dentist* OR (allied W/2 profession*))) | 1,594,600 |
| 1. #10 OR #11 | 3,294,466 |
| 1. #1 AND #9 AND #12 | 1,552 |
| 1. Timeframe between 2020 – 2023 | 548 |

**8. Business Source Complete (EBSCO)**

| 1. (champion*) | 43,222 |
| --- | --- |
| 1. DE "PROFESSIONAL relationships" OR DE "COWORKER relationships" | 3,619 |
| 1. DE "LEADERSHIP" | 80,494 |
| 1. S2 AND S3 | 226 |
| 1. S1 OR S4 | 43,448 |
| 1. (ebp or ebm or ebn or cpg* or best practice* or (evidence n2 practice*)) | 53,683 |
| 1. (guideline* n2 (implement* or adher*)) | 2,085 |
| 1. (quality n1 (improv* or manag*)) | 67,406 |
| 1. (research n2 (use or utili* or adopt* or implement* or disseminat* or uptake or transfer* or translat* or support)) | 35,108 |
| 1. (knowledge n2 (use or utili* or adopt* or implement* or disseminat* or uptake or transfer* or translat* or support)) | 19,605 |
| 1. (evidence n2 (use or utili* or adopt* or implement* or disseminat* or uptake or transfer* or translat* or support)) | 13,961 |
| 1. ((innovation n2 (adopt* or diffus*)) or (implementation n2 (science* or research)) or (innovation or diffusion) | 453,967 |
| 1. DE "INFORMATION resources management" OR DE "EVIDENCE-based management" | 30,798 |
| 1. DE "QUALITY assurance" | 9,018 |
| 1. DE "DIFFUSION of innovations" OR DE "INNOVATION adoption" | 11,402 |
| 1. S6 OR S7 OR S8 OR S9 OR S10 OR S11 OR S12 OR S13 OR S14 OR S15 | 654,994 |
| 1. (healthcare OR health n2 care) | 458,475 |
| 1. ( (health care n2 (professional* or personnel* or provider* or occupation*)) ) OR ( (healthcare n2 (professional* or personnel* or provider* or occupation*)) ) OR ( nurse* or physician* or dentist* or allied profession* ) | 206,376 |
| 1. (((DE "MEDICAL care") OR (DE "HEALTH facilities")) OR (DE "HEALTH services administrators")) OR (DE "HOSPITALS") OR (DE "MEDICAL offices") | 150,486 |
| 1. (DE "MEDICAL personnel") OR (DE "HOSPITAL personnel") | 23,716 |
| 1. S17 OR S18 OR S19 OR S20 | 616,725 |
| 1. S5 AND S16 AND S21 | 100 |
| 1. Timeframe between 2020 - 2023 | 24 |

**Supplementary File 4**: Data extracted from included studies

The following data were extracted from each included study:

- Author (name, correspondence email)
- Year (data collection [from and to] and publication)
- Country/region where study conducted
- Study design (RCT or cRCT)
- Overall effectiveness of trial (changes to policy, process outcomes i.e. behaviour change achieved or not, or patient outcomes as applicable/reported; reported effect measures and confidence intervals)
- Setting (eg inpatient hospital, community etc)
- Details of champion (how identified, discipline, years of experience)
- Health professionals involved in trial (discipline, number)
- Patient group involved (clinical area (i.e. stroke), number)
- Length of implementation period
- Dose of implementation (number of sessions [classified as 1, 2 or multiple], frequency of follow-up or support [classified as none, ad hoc or regular])
- Implementation target (what recommendation/practice/behaviour)
- Implementation strategies used (mapped to categories of Powell 2015, Waltz 2015)
- Details of training for implementation champion: label given to champion, how many champions/site, length of training (days, hours, weeks), components of training package (i.e modules of training), who delivered training and any training material used.
- Details of support provided to implementation champion

**Supplementary File 5:** Full-text studies not retained and reasons for their exclusion

[1-33] External Facilitation (therefore not ‘champion’)

[34-48] Wrong intervention

[12, 22, 27, 35, 49] Duplicate

[49-79]Wrong Study Design

[80, 81]Wrong setting

[82-84]Wrong Patient Population

[85-87]Wrong Outcomes

[88]Wrong Indication

1. Ansari, M., et al., Improving guideline adherence: a randomized trial evaluating strategies to increase beta-blocker use in heart failure. Circulation, 2003. 107(22): p. 2799-804.

2. Calo, W.A., et al., Coaching primary care clinics for HPV vaccination quality improvement: Comparing in-person and webinar implementation. Transl Behav Med, 2019. 9(1): p. 23-31.

3. Chinman, M., et al., Testing implementation support for evidence-based programs in community settings: a replication cluster-randomized trial of Getting To Outcomes(R). Implement Sci, 2018. 13(1): p. 131.

4. Chinman, M., et al., Using Getting To Outcomes to facilitate the use of an evidence-based practice in VA homeless programs: a cluster-randomized trial of an implementation support strategy. Implement Sci, 2017. 12(1): p. 34.

5. Dobbins, M., et al., A randomized controlled trial evaluating the impact of knowledge translation and exchange strategies. Implement Sci, 2009. 4: p. 61.

6. Dobbins, M., et al., A description of a knowledge broker role implemented as part of a randomized controlled trial evaluating three knowledge translation strategies. Implement Sci, 2009. 4: p. 23.

7. Gustafson, D.H.Q., A. R.; Robinson, J. M.; Ford, J. H., 2nd; Pulvermacher, A.; French, M. T.; McConnell, K. J.; Batalden, P. B.; Hoffman, K. A.; McCarty, D., Which elements of improvement collaboratives are most effective? A cluster-randomized trial. Addiction, 2013. 108: p. 1145-1157.

8. Lemelin, J., W. Hogg, and N. Baskerville, Evidence to action: a tailored multifaceted approach to changing family physician practice patterns and improving preventive care. CMAJ, 2001. 164(6): p. 757-63.

9. Jaen, C.R., et al., Methods for evaluating practice change toward a patient-centered medical home. Ann Fam Med, 2010. 8 Suppl 1(Suppl 1): p. S9-20; S92.

10. Kinley, J., et al., The effect of using high facilitation when implementing the Gold Standards Framework in Care Homes programme: a cluster randomised controlled trial. Palliat Med, 2014. 28(9): p. 1099-109.

11. Leathers, S.J., et al., The Effect of a Change Agent on Use of Evidence-Based Mental Health Practices. Adm Policy Ment Health, 2016. 43(5): p. 768-782.

12. Parchman, M.L., et al., A randomized trial of practice facilitation to improve the delivery of chronic illness care in primary care: initial and sustained effects. Implement Sci, 2013. 8: p. 93.

13. Modell, M., et al., A multidisciplinary approach for improving services in primary care: randomised controlled trial of screening for haemoglobin disorders. BMJ, 1998. 317(7161): p. 788-91.

14. Mold, J.W., et al., Implementation of evidence-based preventive services delivery processes in primary care: an Oklahoma Physicians Resource/Research Network (OKPRN) study. J Am Board Fam Med, 2008. 21(4): p. 334-44.

15. Mold, J.W., et al., Implementing asthma guidelines using practice facilitation and local learning collaboratives: a randomized controlled trial. Ann Fam Med, 2014. 12(3): p. 233-40.

16. Pattinson, R.C., et al., Implementation of kangaroo mother care: a randomized trial of two outreach strategies. Acta Paediatr, 2005. 94(7): p. 924-7.

17. Quanbeck, A., et al., A randomized matched-pairs study of feasibility, acceptability, and effectiveness of systems consultation: a novel implementation strategy for adopting clinical guidelines for Opioid prescribing in primary care. Implement Sci, 2018. 13(1): p. 21.

18. Byng, R., et al., Exploratory cluster randomised controlled trial of shared care development for long-term mental illness. Br J Gen Pract, 2004. 54(501): p. 259-66.

19. Meropol, S.B., et al., Practice-tailored facilitation to improve pediatric preventive care delivery: a randomized trial. Pediatrics, 2014. 133(6): p. e1664-75.

20. Salbach, N.M., et al., Facilitated interprofessional implementation of a physical rehabilitation guideline for stroke in inpatient settings: process evaluation of a cluster randomized trial. Implement Sci, 2017. 12(1): p. 100.

21. Shaw, E.K., et al., Effects of facilitated team meetings and learning collaboratives on colorectal cancer screening rates in primary care practices: a cluster randomized trial. Ann Fam Med, 2013. 11(3): p. 220-8, S1-8.

22. Due, T.D., et al., The effectiveness of a semi-tailored facilitator-based intervention to optimise chronic care management in general practice: a stepped-wedge randomised controlled trial. BMC Fam Pract, 2014. 15: p. 65.

23. Tjia, J., et al., Dissemination of Evidence-Based Antipsychotic Prescribing Guidelines to Nursing Homes: A Cluster Randomized Trial. J Am Geriatr Soc, 2015. 63(7): p. 1289-98.

24. van Beurden, I., et al., Involvement of general practitioners in managing alcohol problems: a randomized controlled trial of a tailored improvement programme. Addiction, 2012. 107(9): p. 1601-11.

25. Williams, L., et al., A cluster-randomised quality improvement study to improve two inpatient stroke quality indicators. BMJ Qual Saf, 2016. 25(4): p. 257-64.

26. Yano, E.M., et al., Targeting primary care referrals to smoking cessation clinics does not improve quit rates: implementing evidence-based interventions into practice. Health Serv Res, 2008. 43(5 Pt 1): p. 1637-61.

27. Engels, Y., et al., The effects of a team-based continuous quality improvement intervention on the management of primary care: a randomised controlled trial. Br J Gen Pract, 2006. 56(531): p. 781-7.

28. Aspy, C.B., et al., Improving mammography screening using best practices and practice enhancement assistants: an Oklahoma Physicians Resource/Research Network (OKPRN) study. J Am Board Fam Med, 2008. 21(4): p. 326-33.

29. Cockburn, J., et al., Randomised trial of three approaches for marketing smoking cessation programmes to Australian general practitioners. BMJ, 1992. 304(6828): p. 691-4.

30. Dietrich, A.J., et al., Cancer: improving early detection and prevention. A community practice randomised trial. BMJ, 1992. 304(6828): p. 687-91.

31. Eriksson, L., et al., Lessons learned from stakeholders in a facilitation intervention targeting neonatal health in Quang Ninh province, Vietnam. BMC Pregnancy Childbirth, 2013. 13: p. 234.

32. Frijling, B.D., et al., Multifaceted support to improve clinical decision making in diabetes care: a randomized controlled trial in general practice. Diabet Med, 2002. 19(10): p. 836-42.

33. Harris, M.F., et al., Implementing guidelines to routinely prevent chronic vascular disease in primary care: the Preventive Evidence into Practice cluster randomised controlled trial. BMJ Open, 2015. 5(12): p. e009397.

34. Bentz, C.J., et al., Provider feedback to improve 5A's tobacco cessation in primary care: a cluster randomized clinical trial. Nicotine Tob Res, 2007. 9(3): p. 341-9.

35. Brown, C.H., et al., Evaluation of two implementation strategies in 51 child county public service systems in two states: results of a cluster randomized head-to-head implementation trial. Implement Sci, 2014. 9: p. 134.

36. Glisson, C., et al., Randomized trial of the Availability, Responsiveness and Continuity (ARC) organizational intervention for improving youth outcomes in community mental health programs. J Am Acad Child Adolesc Psychiatry, 2013. 52(5): p. 493-500.

37. Lobo, C.M., et al., Improving quality of organizing cardiovascular preventive care in general practice by outreach visitors: a randomized controlled trial. Prev Med, 2002. 35(5): p. 422-9.

38. Margolis, P.A., et al., Practice based education to improve delivery systems for prevention in primary care: randomised trial. BMJ, 2004. 328(7436): p. 388.

39. Palter, V.N., et al., Peer coaching to teach faculty surgeons an advanced laparoscopic skill: A randomized controlled trial. Surgery, 2016. 160(5): p. 1392-1399.

40. Peterson, D.J., et al., COMMUNITY TRANSLATION OF FALL PREVENTION INTERVENTIONS: THE METHODS AND PROCESS OF A RANDOMIZED TRIAL. 2015. 43(8): p. 1005-1018.

41. Traynor, R., K. DeCorby, and M. Dobbins, Knowledge brokering in public health: a tale of two studies. Public Health, 2014. 128(6): p. 533-44.

42. Williams, N.J., et al., Mechanisms of Change in the ARC Organizational Strategy: Increasing Mental Health Clinicians' EBP Adoption Through Improved Organizational Culture and Capacity. Adm Policy Ment Health, 2017. 44(2): p. 269-283.

43. Johnson, H., et al., Implementation of a complex intervention to improve care for patients whose situations are clinically uncertain in hospital settings: A multi-method study using normalisation process theory. PLoS One, 2020. 15(9): p. e0239181.

44. Keck, J.W., et al., Primary Care Cluster RCT to Increase Diabetes Prevention Program Referrals. Am J Prev Med, 2020. 59(1): p. 79-87.

45. Ma, L., et al., The third Intensive Care Bundle with Blood Pressure Reduction in Acute Cerebral Haemorrhage Trial (INTERACT3): an international, stepped wedge cluster randomised controlled trial. Lancet, 2023. 402(10395): p. 27-40.

46. Pastva, A.M., et al., Movement Matters, and So Does Context: Lessons Learned From Multisite Implementation of the Movement Matters Activity Program for Stroke in the Comprehensive Postacute Stroke Services Study. Arch Phys Med Rehabil, 2021. 102(3): p. 532-542.

47. Paul, C.L., et al., Care to Quit: a stepped wedge cluster randomised controlled trial to implement best practice smoking cessation care in cancer centres. Implement Sci, 2021. 16(1): p. 23.

48. Rawl, S.M., et al., Computer-tailored intervention increases colorectal cancer screening among low-income African Americans in primary care: Results of a randomized trial. Prev Med, 2021. 145: p. 106449.

49. Holdsworth, E., R. Ryall, and E. Greenwood, 783 ADVANCE CARE PLANNING IN A LARGE TEACHING HOSPITAL EMERGING FROM THE COVID-19 PANDEMIC: A QUALITY IMPROVEMENT PROJECT. Age & Ageing, 2022. 51: p. 18.

50. Kelly, J.A., et al., Bridging the gap between the science and service of HIV prevention: transferring effective research-based HIV prevention interventions to community AIDS service providers. Am J Public Health, 2000. 90(7): p. 1082-8.

51. McBride, P., et al., Improving prevention systems in primary care practices: the Health Education and Research Trial (HEART). J Fam Pract, 2000. 49(2): p. 115-25.

52. Stange, K.C., et al., Sustainability of a practice-individualized preventive service delivery intervention. Am J Prev Med, 2003. 25(4): p. 296-300.

53. Agarwal, A., et al., Protocol for a cluster randomised trial in Madhya Pradesh, India: community health promotion and medical provision and impact on neonates (CHAMPION2); and support to rural India's public education system and impact on numeracy and literacy scores (STRIPES2). Trials, 2020. 21(1): p. 569.

54. Anand, P., et al., Dissemination of Best Practices in Preterm Care Through a Novel Mobile Phone-Based Interactive e-Learning Platform. Indian J Pediatr, 2021. 88(11): p. 1068-1074.

55. Bernstein, K., et al., Creating a Culture of Breastfeeding Support and Continuity of Care in Central Illinois. Health Promot Pract, 2022. 23(1_suppl): p. 108S-117S.

56. Biehl, R. and A. Efre, Improving human papilloma virus vaccination rates among adolescents. J Am Assoc Nurse Pract, 2023. 35(10): p. 642-645.

57. Bradley, K., et al., Let's CHAT (community health approaches to) dementia in Aboriginal and Torres Strait Islander communities: protocol for a stepped wedge cluster randomised controlled trial. BMC Health Serv Res, 2020. 20(1): p. 208.

58. Cullen, L., et al., Evidence-Based Practice Change Champion Program Improves Quality Care. J Nurs Adm, 2020. 50(3): p. 128-134.

59. Dupuis, L.L., et al., Readiness to Implement Symptom Management Care Pathways in Pediatric Cancer. Res Sq, 2020.

60. Edge, N.B., Breaking the Cycle: Care Coordination Interventions and Sickle Cell Readmissions. Prof Case Manag, 2022. 27(1): p. 12-18.

61. Ehlers, S.L., et al., Real-World Implementation of Best-Evidence Cancer Distress Management: Truly Comprehensive Cancer Care. J Natl Compr Canc Netw, 2023. 21(6): p. 627-635.

62. Fasugba, O., et al., Evaluating remote facilitation intensity for multi-national translation of nurse-initiated stroke protocols (QASC Australasia): a protocol for a cluster randomised controlled trial. Implement Sci, 2023. 18(1): p. 2.

63. Gaynes, B.N., et al., The Sub-Saharan Africa Regional Partnership (SHARP) for Mental Health Capacity-Building Scale-Up Trial: Study Design and Protocol. Psychiatr Serv, 2021. 72(7): p. 812-821.

64. Hamilton, W., et al., Aromatherapy: Use of Essential Oils to Decrease Pain, Anxiety, and Nausea in Acute Care. Medsurg Nursing, 2022. 31(2): p. 110-113.

65. Huffstetler, A.N., et al., Practice facilitation to promote evidence-based screening and management of unhealthy alcohol use in primary care: a practice-level randomized controlled trial. BMC Fam Pract, 2020. 21(1): p. 93.

66. Isaacs, D., et al., Technology Integration: The Role of the Diabetes Care and Education Specialist in Practice. Diabetes Educ, 2020. 46(4): p. 323-334.

67. Iwuji, C., et al., Optimised electronic patient records to improve clinical monitoring of HIV-positive patients in rural South Africa (MONART trial): study protocol for a cluster-randomised trial. BMC Infect Dis, 2021. 21(1): p. 1266.

68. Joag, K., et al., Atmiyata, a community-led intervention to address common mental disorders: Study protocol for a stepped wedge cluster randomized controlled trial in rural Gujarat, India. Trials, 2020. 21(1): p. 212.

69. Liu, M., et al., The ACCELERATE Plus (assessment and communication excellence for safe patient outcomes) Trial Protocol: a stepped-wedge cluster randomised trial, cost-benefit analysis, and process evaluation. BMC Nurs, 2023. 22(1): p. 275.

70. Phillips, S.J., et al., Improving stroke care in Nova Scotia, Canada: a population-based project spanning 14 years. BMJ Open Qual, 2021. 10(3).

71. Ridgway, J.P., et al., POWER Up-Improving pre-exposure prophylaxis (PrEP) uptake among Black cisgender women in the Southern United States: Protocol for a stepped-wedge cluster randomized trial (SW-CRT). PLoS One, 2023. 18(5): p. e0285858.

72. Rihari-Thomas, J., et al., Assessment and communication excellence for safe patient outcomes (ACCELERATE): A stepped-wedge cluster randomised trial protocol. Collegian, 2022. 29(5): p. 799-805.

73. Rosen, C.S., et al., Targeted Assessment and Context-Tailored Implementation of Change Strategies (TACTICS) to increase evidence based psychotherapy in military behavioral health clinics: Design of a cluster-randomized stepped-wedge implementation study. Contemp Clin Trials, 2020. 93: p. 106008.

74. Sapag, J.C., et al., Reducing stigma toward mental illness and substance use issues in primary health care in Chile: Protocol of a cluster controlled trial study. Front Psychiatry, 2022. 13: p. 1083042.

75. Stiell, I., et al., LO04: Decreasing emergency department length of stay for patients with acute atrial fibrillation and flutter: a cluster-randomized trial. Canadian Journal of Emergency Medicine, 2020. 22: p. S7 - S8.

76. Stokes, T., et al., Implementation of the Diabetes Community Exercise and Education Programme (DCEP) for the management of type 2 diabetes: qualitative process evaluation. BMJ Open, 2022. 12(5): p. e059853.

77. Szmuilowicz, E., et al., The PACT Project: Feasibility of a Multidisciplinary, Multi-Faceted Intervention to Promote Goals of Care Conversations. Am J Hosp Palliat Care, 2024. 41(4): p. 355-362.

78. Weir, A., et al., Strategies for facilitating the delivery of cluster randomized trials in hospitals: A study informed by the CFIR-ERIC matching tool. Clin Trials, 2021. 18(4): p. 398-407.

79. Wilkinson, K., et al., Implementation of training to improve communication with disabled children on the ward: A feasibility study. Health Expect, 2021. 24(4): p. 1433-1442.

80. Murray, M.E., et al., Is more better? Examining whether enhanced consultation/coaching improves implementation. Am J Orthopsychiatry, 2018. 88(3): p. 376-385.

81. Moyo, P., et al., Effect of a Video-Assisted Advance Care Planning Intervention on End-of-Life Health Care Transitions Among Long-Stay Nursing Home Residents. J Am Med Dir Assoc, 2022. 23(3): p. 394-398.

82. Champion, V.L., et al., Comparative Effectiveness of 2 Interventions to Increase Breast, Cervical, and Colorectal Cancer Screening Among Women in the Rural US: A Randomized Clinical Trial. JAMA Netw Open, 2023. 6(4): p. e2311004.

83. Le Goff, D., et al., Innovative cardiovascular primary prevention population-based strategies: a 2-year hybrid type 1 implementation randomised control trial (RCT) which evaluates behavioural change conducted by community champions compared with brief advice only from the SPICES project (scaling-up packages of interventions for cardiovascular disease prevention in selected sites in Europe and sub-Saharan Africa). BMC Public Health, 2021. 21(1): p. 1422.

84. Soler-Font, M., et al., Process evaluation of a complex workplace intervention to prevent musculoskeletal pain in nursing staff: results from INTEVAL_Spain. BMC Nurs, 2021. 20(1): p. 189.

85. Maunder, R.G., et al., Randomized trial of personalized psychological feedback from a longitudinal online survey and simultaneous evaluation of randomized stepped wedge availability of in-person peer support for hospital staff during the COVID-19 pandemic. Gen Hosp Psychiatry, 2023. 84: p. 31-38.

86. Stanley, B., et al., Zero suicide implementation-effectiveness trial study protocol in outpatient behavioral health using the A-I-M suicide prevention model. Contemp Clin Trials, 2021. 100: p. 106224.

87. Vaughan, C.P., et al., A cluster-randomized trial of two implementation strategies to deliver audit and feedback in the EQUIPPED medication safety program. Acad Emerg Med, 2023. 30(4): p. 340-348.

88. Sabo, R.T., et al., Low-Intensity Intervention Supports Diabetes Registry Implementation: A Cluster-Randomized Trial in the Ambulatory Care Outcomes Research Network (ACORN). J Am Board Fam Med, 2020. 33(5): p. 728-735.

**Supplementary File 6**: PEDro scale scores to assess the of risk of bias (n=15)

|  | Random Allocation | Allocation Concealed | Similar at baseline regarding prognostic measures | Subject Blinding | Therapist Blinding | Assessor Blinding | More than 85% of key outcomes | “Intention to treat” | Results of between-group statistical comparisons | Point measures and measures of variability | Total score /10 |
| --- | --- | --- | --- | --- | --- | --- | --- | --- | --- | --- | --- |
| Acolet 2011 [42] | Yes | Yes | Yes | Yes | No | Yes | Yes | Yes | Yes | Yes | 9 |
| Ayieko 2011 [34] | Yes | No | Yes | Yes | No | No | Yes | Yes | Yes | Yes | 7 |
| Bailey 2021 [38] | Yes | No | Yes | No | No | No | Yes | Yes | Yes | Yes | 6 |
| Bunce 2020 [35] | Yes | No | Yes | Yes | No | No | Yes | Yes | Yes | Yes | 7 |
| Cadilhac 2022 [36] | No | No | Yes | Yes | Yes | Yes | Yes | Yes | Yes | Yes | 8 |
| Duclos 2022 [45] | Yes | Yes | Yes | Yes | No | No | Yes | Yes | Yes | Yes | 8 |
| Johnston 2007 [39] | Yes | No | Yes | No | No | No | Yes | Yes | Yes | Yes | 6 |
| Llewelyn 2023 [46] | Yes | Yes | Yes | No | No | No | Yes | Yes | Yes | Yes | 7 |
| Middleton 2019 [44] | Yes | Yes | Yes | Yes | No | Yes | Yes | Yes | Yes | Yes | 9 |
| Mudge 2022 [40] | Yes | No | Yes | No | No | No | Yes | Yes | Yes | Yes | 6 |
| Puchalski-Ritchie 2021 [41] | Yes | No | Yes | No | No | Yes | Yes | Yes | Yes | Yes | 7 |
| Resnick 2021 [47] | Yes | No | Yes | Yes | No | No | Yes | Yes | Yes | Yes | 7 |
| Resnick 2021 [43] | Yes | No | Yes | Yes | No | Yes | Yes | Yes | Yes | Yes | 8 |
| Rycroft-Malone 2012 [37] | Yes | Yes | Yes | Yes | No | No | Yes | Yes | Yes | Yes | 8 |
| Seers 2018 [13] | Yes | Yes | Yes | Yes | No | Yes | Yes | Yes | Yes | Yes | 9 |
